# Alterations of lung microbial communities in obese allergic asthma and metabolic potential

**DOI:** 10.1371/journal.pone.0256848

**Published:** 2021-10-28

**Authors:** Jongan Lee, Sung-hee Lee, Gyo Jeong Gu, Ji hyun Choi, Kyu-Tae Jeong, Jeom-Kyu Lee, Seung Hyun Kim

**Affiliations:** Division of Allergy and Respiratory Diseases Research, Department of Chronic Disease Convergence Research, National Institute of Health, Korea Disease Control and Prevention Agency, Cheongju, Republic of Korea; University of California, Davis, UNITED STATES

## Abstract

In recent years, there has been a rapid increase in microbiome studies to explore microbial alterations causing disease status and unveil disease pathogenesis derived from microbiome environmental modifications. Convincing evidence of lung microbial changes involving asthma has been collected; however, whether lung microbial changes under obesity leads to severe asthma in a state of allergen exposure has not been studied sufficiently. Here, we measured bacterial alterations in the lung of an allergen mouse model induced by a high fat diet (HFD) by using 16S rRNA gene sequencing. A total of 33 pathogen‑free 3‑week‑old male C57BL/6 mice were used, and they divided randomly into two groups. The Chow diet (n = 16) and high fat diet (n = 17) was administrated for 70 days. Mice were sensitized with PBS or *Dermatophagoides pteronyssinus* extract (Der.p), and concentration levels of total IgE and Der.p-IgE in the blood were measured to quantify immune responses. Although there were no meaningful differences in bacterial species richness in the HFD mouse group, momentous changes of bacterial diversity in the HFD mouse group were identified after the mouse group was exposed to allergens. At a genus level, the fluctuations of taxonomic relative abundances in several bacteria such as *Ralstonia*, *Lactobacillus*, *Bradyrhizobium*, *Gaiella*, *PAC001932_g*, *Pseudolabrys*, and *Staphylococcus* were conspicuously observed in the HFD mouse group exposed to allergens. Also, we predicted metabolic signatures occurring under microbial alterations in the Chow group versus the Chow group exposed to allergens, as well as in the HFD mouse group versus the HFD group exposed to allergens. We then compared their similarities and differences. Metabolic functions associated with macrophages such as propanoate metabolism, butanoate metabolism, and glycine-serine-threonine metabolism were identified in the HFD group versus the Chow group. These results provide new insights into the understanding of a microbiome community of obese allergic asthma, and shed light on the functional roles of lung microbiota inducing the pathogenesis of severe asthma.

## Introduction

Asthma is a heterogeneous disorder accompanied by chronic inflammations, worsening functions, and remodeling in the airway [1]. Effects of comorbidities on clinical asthma such as obesity, metabolic syndromes, diabetes mellitus, cardiovascular diseases, and mental diseases are still unclear [2]. Among such diseases, obesity is considered a critical risk factor for asthma. An increased prevalence of asthma was discovered in obese versus lean subjects in epidemiologic studies, and the correlation between obesity and asthma was identified regardless of ethnicity, age, and sex [3–5]. Obesity not only augments the incidence of asthma but also increases the severity of asthma [6]. For instance, participants showing current severe asthma (1.4%) among 32,644 adults were associated with their BMI classes: overweight (25.0–29.9kg/m^2^), obesity class I and II (30.0–39.9kg/m^2^), and obesity class III (> = 40.0kg/m^2^) with odd ratios of 1.36, 1.50, and 3.70 [7].

Arrieta and colleagues explored gut microbiota dysbiosis, and the decreased abundances of *Lachnospira*, *Veillonella*, *Faecalibacterium*, and *Rothia* were identified in children at risk of asthma [8]. Furthermore, specific bacteria such as *Haemophilus*, *Streptococcus*, and *Moraxella* were associated with an occurrence of asthma exacerbation and with asthma progression [9, 10]. Emerging evidence suggests that alterations in dietary patterns affect gut microbiota and the bi-directional cross-talk between gut and lung, and their influences are implicated in several respiratory diseases such as allergy, asthma, and cystic fibrosis [11]. Alterations in lung microbial compositions influence gut microbiota. For instance, the influenza virus-infected respiratory tract in mice resulted in an augmentation of *Enterobacteriaceae* and a concurrent reduction of *Lactobacilli* in the intestinal microbiota [12]. The imbalance in mice lung microbiota under Lipopolysaccharide (LPS) exposure affected gut microbiota dysbiosis because of bacterial translocation from the lung into the bloodstream [13]. This article also elucidates interactions of microorganisms between the gut and lungs. For instance, microbiota from the gastrointestinal tract (GIT) could permeate the lungs through the airway [14]. The microbial imbalance in the lung leads to susceptibility to asthma and aggravates asthma symptoms [15]. Bronchial microbiota enriched in a *Klebsiella* species were detected in severe asthma patients, compared with healthy subjects. Significant differences in the abundance of both *Actinobacteria* and *Gammaproteobacteria* were found in severe asthmatics versus mild-to-moderate asthmatics [16]. In addition, increases of both *Proteobacteria* and *Firmicutes* in severe asthmatics were identified, compared with healthy controls, and corresponding decreases of both *Fusobacteria* and *Bacteroidetes* were observed in the airway [17]. These studies suggest that changes in both lung and gut microbiota are significantly connected with asthma pathogenesis and severity; however, whether lung microbiota conditions under obesity could be altered by allergen exposure that gives rise to severe asthma has not been studied sufficiently.

In this study, we explored whether obese allergic asthma is derived from allergen exposure, which not only causes the dysfunctions of the immune system in the lung but also affects lung microbiome surroundings. For this purpose, the *Dermatophagoides pteronyssinus* (Der.p) extract sensitization and challenge was performed in both Chow and HFD mice, and lung tissues were harvested 70 days later. Then, high-throughput 16S rRNA gene sequencing was performed from lung tissues. Finally, we carried out an alpha diversity analysis to evaluate bacterial species richness and diversity; conducted a beta diversity analysis to uncover similarities between samples; and performed functional prediction based on 16S rRNA genes sequencing.

## Materials and methods

### Animals

All experimental procedures were approved by the Institutional Animal Care and Use Committee of Korea Disease Control and Prevention Agency (approval number: KCDC-083-18-2A). Specific pathogen‑free 3‑week‑old male C57BL/6 mice, purchased from Orient Bio Inc. (Seoul, Korea), were used after going through a process of quarantine and acclimatization for one week. C57BL/6 male mice were chosen due to their increased susceptibility for developing signs of obesity by high fat diet compared with other mouse strains and because of female mice are resistant to the induction of obesity by high fat diet [18]. Mice were housed in a disposable IVC rodent cage system (Innovive, San Diego, USA) under environmentally-controlled conditions (a 12/12-h light/dark cycle, a temperature of 21 ± 2 °C, and a relative humidity of 50 ± 20%) in an animal research facility. The experiment was conducted between February and December 2018.

### Experimental procedures

#### Pretreatment of antibiotics

Before the establishment of the experimental model, we depleted the microbiota of conventional mice with a cocktail of four antibiotics (4 mg/mL of ampicillin, 4 mg/mL of vancomycin, 4 mg/mL of neomycin, and 4 mg/mL of metronidazole) via the drinking water for four days [19, 20]. The drinking water was replaced with fresh water with antibiotic mixtures every day.

#### Diet-induced obesity

In the former set, mice were divided into two groups with similar body weights. Mice were fed with either a normal diet (Chow), 10% kcal fat 35% sucrose diet (D12450B; Research Diet Inc., NJ, USA) (n = 16), or a high-fat diet (HFD), 45% kcal fat 17% sucrose diet (D12451; Research Diet Inc., NJ, USA) (n = 17) for 70 days. The contents of these diets shown in S1 Table. Mice were weighed using a digital scale (ED3202S-CW Precision Balances, Satorius) with their weights recorded on a weekly basis. After 42 days of feeding the mice were fasted for 14 h by placing them in newly made-up cages without food but with access to water and challenged with 2g glucose/kg via intraperitoneal injection [21]. Immediately preceding glucose injection, and at 15, 30, 60, and 120 min post-injection, glucose levels on the blood from the tail vain were measured by glucometer (Accu-Chek Active, Roche).

#### HDM driven allergic asthma

**Fig 1:**
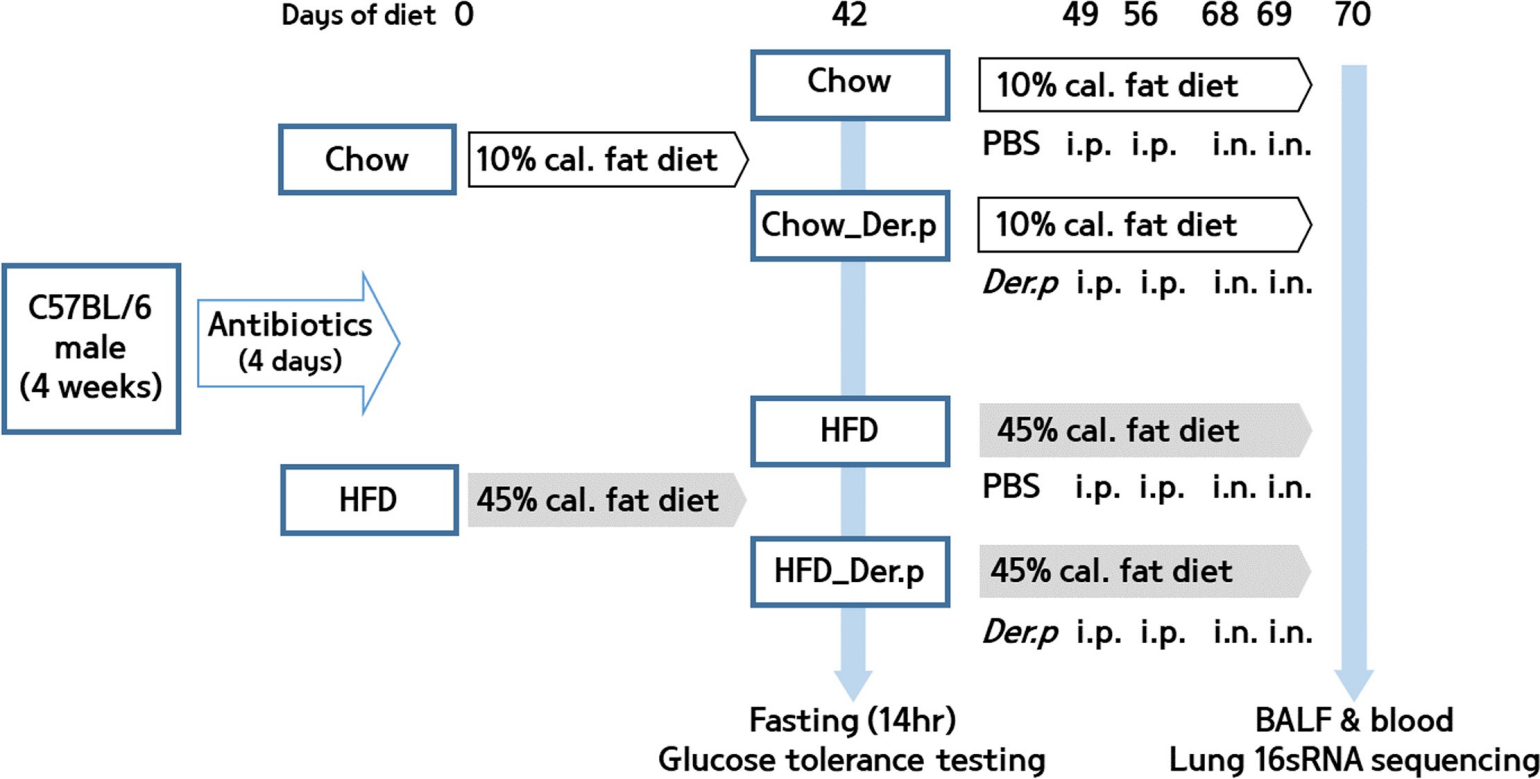
Overview of the experiment. From 4 weeks of age, C57BL/6 male mice (n = 33) were pretreated with a cocktail of antibiotics for 4 days, and separated into 2 treatment groups and fed either a normal diet (Chow, n = 16) or high fat diet (HFD, n = 17). A glucose tolerance test (GTT) was performed after mice had been fed either diet for 42 days. From 49 days, the two diet groups were separated into 2 groups respectively, a normal diet-fed group with phosphate-buffered saline (PBS) sensitization and challenge (Chow, n = 9), a normal diet-fed group with Der.p sensitization and challenge (Chow_Der.p, n = 7), a high fat diet-fed group with PBS sensitization and challenge (HFD, n = 7), and a high fat diet-fed group with Der.p sensitization and challenge (HFD_Der.p group, n = 10). After 70 days of diet, blood and lung tissues were collected.

In the latter set, the two diet groups were divided into two groups with similar weights to induce asthma caused by house dust mites: a normal diet-fed group with phosphate-buffered saline (PBS) sensitization and challenge (Chow, n = 9), a normal diet-fed group with Der.p sensitization and challenge (Chow_Der.p, n = 7), a high fat diet-fed group with PBS sensitization and challenge (HFD, n = 7), and a high fat diet-fed group with Der.p sensitization and challenge (HFD_Der.p group, n = 10). On days 49 and 56, mice were sensitized intraperitoneally with either PBS or 50 µg of *Dermatophagoides pteronyssinus* (Der.p) crude extract (Prolagen, Seoul, Korea) dissolved in 200 µL of PBS. Purified house dust mite extract from *Dermatophagoides pteronyssinus* (Der.p; lot number ACPF-01; Prolagen, Seoul, Korea) with a known content of *Der*. *p 1*(4.72μg/mg dry weight) and of the endotoxin level (2526.67 EU/mg) was used in these experiments. The doses of Der.p used in this manuscript refer to the amount of supplied extract product. On days 68 and 69, mice were challenged intranasally with either PBS or 10 μg of Der.p dissolved in 40 μL of PBS under intraperitoneal pentobarbital sodium (0.2 mg/kg) anesthesia [22, 23]. The experimental overview is shown in Fig 1.

#### Characterization of the obese allergic asthma models

On the day after a final Der.p challenge, mice were anesthetized with an intraperitoneal injection of pentobarbital sodium (0.5 mg/kg), tracheotomized using an 22-gauge blunt needle, and then mechanically ventilated at 200 breaths/min using a FlexiVent Fx1 computer-controlled small animal ventilator (SCIREQ, Montreal, Canada). Airway responsiveness is represented as the average of the three peak measurements for each mice, obtained at incremental methacholine (Sigma, St. Louis, USA) doses (12.5, 25, and 50 mg/ml). Blood was obtained by Orbital blood collection, and serum levels of total IgE and Der.p specific IgE were measured using ELISA (BD Biosciences, San Diego, CA, USA). Lung tissues from euthanized mice were obtained using sterile scissors and tweezers for animal surgery and kept at -70 ℃ for 16S rRNA gene sequencing.

### Additional experimental procedures

#### Obese allergic asthma models

For the characterization of the obese allergic asthma model, male C57BL/6 mice were used. As previously described, we pretreated a cocktail of four antibiotics via the drinking water for four days and fed with either a normal diet (Chow) or a high-fat diet (HFD) for 70 days. From 49 days, a normal diet-fed group with phosphate-buffered saline (PBS) sensitization and challenge (Chow, n = 4), a normal diet-fed group with Der.p sensitization and challenge (Chow_Der.p, n = 4), a high fat diet-fed group with PBS sensitization and challenge (HFD, n = 4), and a high fat diet-fed group with Der.p sensitization and challenge (HFD_Der.p group, n = 4). After 70 days of diet, Lung tissues were collected.

#### Histologic and flow cytometry analysis

Left lung lobes were excised and fixed in paraformaldehyde postmortem. Tissue blocks were embedded in paraffin, and the slide were stained with Hematoxylin and eocin(H&E) for evaluation of lung inflammation (Maxbiotech, Korea). Superior lobes were dissociated into single cell suspension by using the Lung Dissociation kit. Briefly, the lung tissue was cut into small pieces with scissors, transferred into C-tubes (Miltenyi, Auburn, CA), and processed in digestion buffer (1 mg/ml of Collagenase D and 0.1 mg/ml DNase I, both from Roche, Indianapolis, IN, in Hanks’ balanced salt solution) and a Gentle MACS dissociator (Miltenyi Biotec Inc., CA), according to the manufacturer’s instructions. Homogenized lungs were passed through 40-μm nylon mesh to obtain a single-cell suspension. The remaining red blood cells were lysed using BD Pharm Lyse (BD Biosciences, San Jose, CA). Cells were stained using the following fluorochrome-conjugated antibodies: PE anti-mouse F4/80 (BM8) (eBioscience), PerCP/Cy5.5 anti-mouse CD45 (30-F11) (BioLegend), anti-FITC anti-mouse CD11c (HL3) (BD pharmingen) and PE-Cy7 anti-mouse CD206 (MR6F3) (Invitrogen). Appropriate isotype controls were also purchased respectively. For each Ab combination, 106 cells were incubated with mAbs for 30min in the dark at 4℃; the cells were than washed and fixed in 1% paraformaldehyde and counted on a BD FACSVerse flow cytometer system (BD Bioscience). Specific MFI was calculated by subtracting the isotype MFI from the Ab-specific MFI. The data analysis was performed using BD FlowJo Software (Tree Star, Ashland, OR). M1, M2 or Alveolar (Av) macrophages were identified as F4/80-positive/CD11c-positive/CD206-negative, F4/80-positive/CD11c-negative/CD206-positive cells or F4/80-positive/CD11c-positive/CD206-positive cells, respectively [24].

### DNA extraction and PCR amplification and 16S rRNA gene sequencing

DNA extraction of lung tissues was performed through the Fast DNA SPIN Kit for Soil (MP Biomedicals Inc., Solon, USA) according to the manufacturer’s instructions. After extracting DNA, PCR amplification was carried out by using a pair of primers to cover the V3-V4 regions of the 16S rRNA gene. A pair of primers (a forward primer: 5’-TCGTCGGCAGCGTCAGATGTGTATAAGAGACAGCCTACGGGNGGCWGCAG-3’; and a reverse primer: 5’-GTCTCGTGGGCTCGGAGATGTGTATAAGAGACAGGACTACHVGGGTATCTAATCC-3’) were used for bacterial amplification. The amplifications were executed based on the following criteria: initial denaturation at 95°C for 3 min, followed by 25 cycles of denaturation at 95°C for 30 sec, primer annealing at 55°C for 30 sec, and extension at 72°C for 30 sec, with a final elongation at 72°C for 5 min. To attach the illumina NexTera barcode, the secondary amplification was carried out by using a pair of primers (a forward primer: 5’-AATGATACGGCGACCACCGAGATCTACACXXXXXXXXTCGTCGGCAGCGTC-3’; and a reverse primer: 5’-CAAGCAGAAGACGGCATACGAGATXXXXXXXXGTCTCGTGGGCTCGG-3’: X represents the barcode). The PCR product was confirmed by using 1% agarose gel electrophoresis and visualized under a Gel Doc system (BioRad, Hercules, CA, USA). The amplified products were purified with the CleanPCR (CleanNA). Equal concentrations of purified products were pooled together and removed short fragments (non-target products) with CleanPCR (CleanNA). The DNA 7500 chip was utilized to estimate the quality and product size through a Bioanalyzer 2100 (Agilent, Palo Alto, CA, USA). Pooling mixed amplicons and sequencing with illumina MiSeq Sequencing system (Illumina, USA) were performed at Chunlab, Inc. (Seoul, Korea).

### Sequencing data processing and taxonomic assignment

Processing raw reads started with quality check and filtering of low quality (<Q25) reads by Trimmomatic ver. 0.32 [25]. After QC pass, paired end sequence data were merged together using fastq_mergepairs command of VSEARCH version 2.13.4 [26] with default parameters. Primers were then trimmed with the alignment algorithm of Myers & Miller [27] at a similarity cut off of 0.8. Non-specific amplicons that do not encode 16S rRNA were detected by nhmmer [28] in HMMER software package ver. 3.2.1 with hmm profiles. Unique re ads were extracted and redundant reads were clustered with the unique reads by derep_fulllength command of VSEARCH [26]. The EzBioCloud 16S rRNA database [29] was used for taxonomic assignment using usearch_global command of VSEARCH [26] followed by more precise pairwise alignment [27]. Chimeric reads were filtered on reads with <97% similarity by reference based chimeric detection using UCHIME algorithm [30] and the non-chimeric 16S rRNA database from EzBioCloud. After chimeric filtering, r reads that are not identified to the species level (with <97% similarity) in the EzBioCloud database were compiled and cluster_fast command [26] was used to perform de-novo clustering to generate additional OTUs. Finally, OTUs with single reads (singletons) are omitted from further analysis.

### Bioinformatics and statistics

A comparative analysis of microbiome taxonomic profiles (MTPs) was fulfilled through BIOiPLUG ChunLab’s bioinformatics cloud platform. Compositional data were normalized using a gene copy number. The Wilcoxon rank-sum test was performed to estimate the differences of taxonomic relative abundances between groups. An alpha diversity analysis was performed to identify species richness and diversity in samples. The alpha diversity refers to the variety within a specific area or habitat, and is generally evaluated by the number of species or evenness. The richness of bacterial species was estimated using Chao1, the abundance-based coverage estimators (ACE), Jackknife, and the number of operational taxonomic units (OTUs). Shannon index, Simpson index, and a phylogenetic index were calculated to uncover bacterial diversity. Also, a beta diversity analysis was conducted to investigate clustering patterns between samples. The beta diversity refers to the comparison of diversity between area or ecosystems, and is commonly quantified as the amount of species change between group. Both the principal coordinate analysis (PCoA) and the unweighted pair group method with arithmetric mean (UPGMA) clustering were executed for a beta diversity analysis. The metagenome functional contents derived from 16S rRNA gene were predicted using a phylogenetic investigation of communities through the reconstruction of unobserved states (PICRUSt). A linear discriminant analysis Effect Size (LEfSe) analysis [31] was then performed for an effect size estimation of each feature, considering statistical significance with p<0.05 and LDA score>2.0.

Data were compared using one-way ANOVA or an unpaired two-way student’s t test of correlated (Spearman’s rank correlation) using the GraphPad Prism 5 for window statistical analysis program. Differences were considered significant with a p-value <0.05.

## Results

### *Dermatophagoides pteronyssinus* (Der.p) crude extract induced allergic response and airway hyperresponsiveness in obese mice

**Fig 2:**
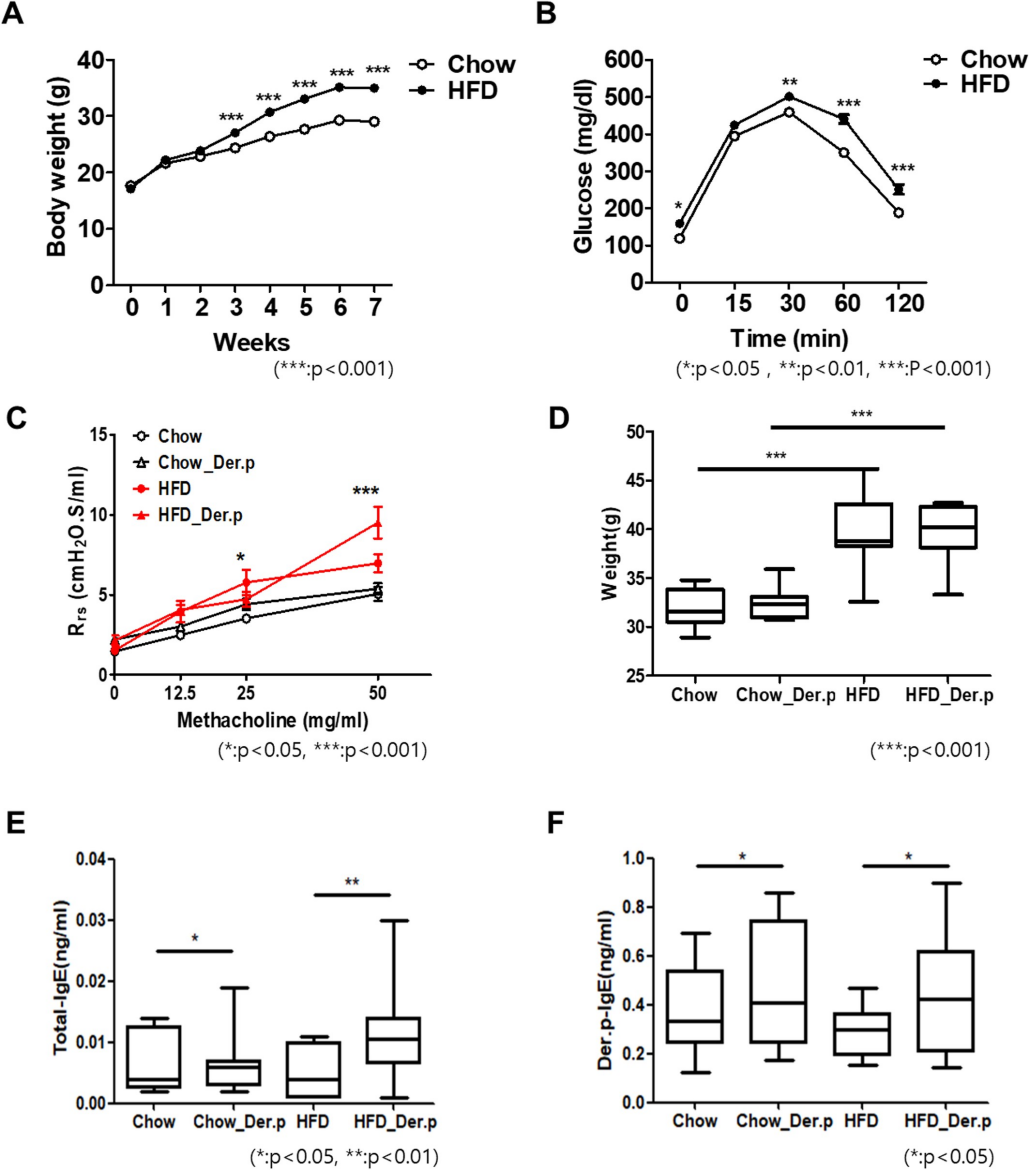
Body weight, IPGTT, AHR, the levels of Der.p-specific IgE, and total IgE in groups. (A) The body weight of High-fat diet (HFD) group (n = 17) gradually increased compared with a normal diet (Chow) group (n = 16). (B) Comparison of blood glucose levels in Chow and HFD group by an IPGTT. (mean ± SEM for 16–17 mice/treatment) (C) The AHR was measured by total resistance of the repiratory system(Rrs) in response to methacholine. (D) Comparison of body weight at 70 days gained in Chow and HFD mice. (E, F) The levels of total-IgE and Der.p specific-IgE in serum. (mean ± SEM for 7–10 mice/treatment) (*p<0.05, **p<0.01, ***p<0.001).

To establish obese allergic asthma model, pathogen-free C57BL/6 male mice were fed with a 45% high-fat diet for 70 days, and as a control group, 10% low-fat diet were used for feeding. Mice were weighed every week, and we found that the weight of high-fat diet group (HFD, n = 17) gradually increased compared with normal diet group (Chow, n = 16) (Fig 2A). As shown in Fig 2B, the HFD group had significantly higher serum fasting glucose levels compared to the Chow group. By the sixth week, the body weight of the HFD group was remarkably lower than the HFD group (p<0.001) (Fig 2D). To induce allergic asthma, mice were sensitized intraperitoneally with Der.p crude extract on days 49 and 56. On days 68 and 69, mice were challenged intranasally with Der.p. The airway hyperresponsiveness (AHR) was increased in HFD group and compared with Chow group and significantly elevated in high-fat diet with the exposure of Der.p. (HFD_Der.p) group compared with HFD group (Fig 2C). Allergic responses were measured by total Immunoglobulin E (IgE) and Der.p-specific IgE in the blood (Fig 2E and 2F). In the both Chow and HFD groups, the concentration levels of Der.p-IgE were increased after allergen exposure (p<0.05). Similarly, the concentration levels of total IgE were increased in the both Chow group (p<0.05) and HFD groups (p<0.01).

We confirmed the structure of lung tissues and the proportion of macrophages in additional experimental set (n = 4 per group). As shown in S1 Fig., the airway inflammatory reaction occurred due to exposure to the allergen, and the changes in the proportion of macrophage phenotypes (M1, M2 and alveolar macrophages) was more significant in the case of obesity.

### Changes of taxonomic relative abundances between groups

**Fig 3:**
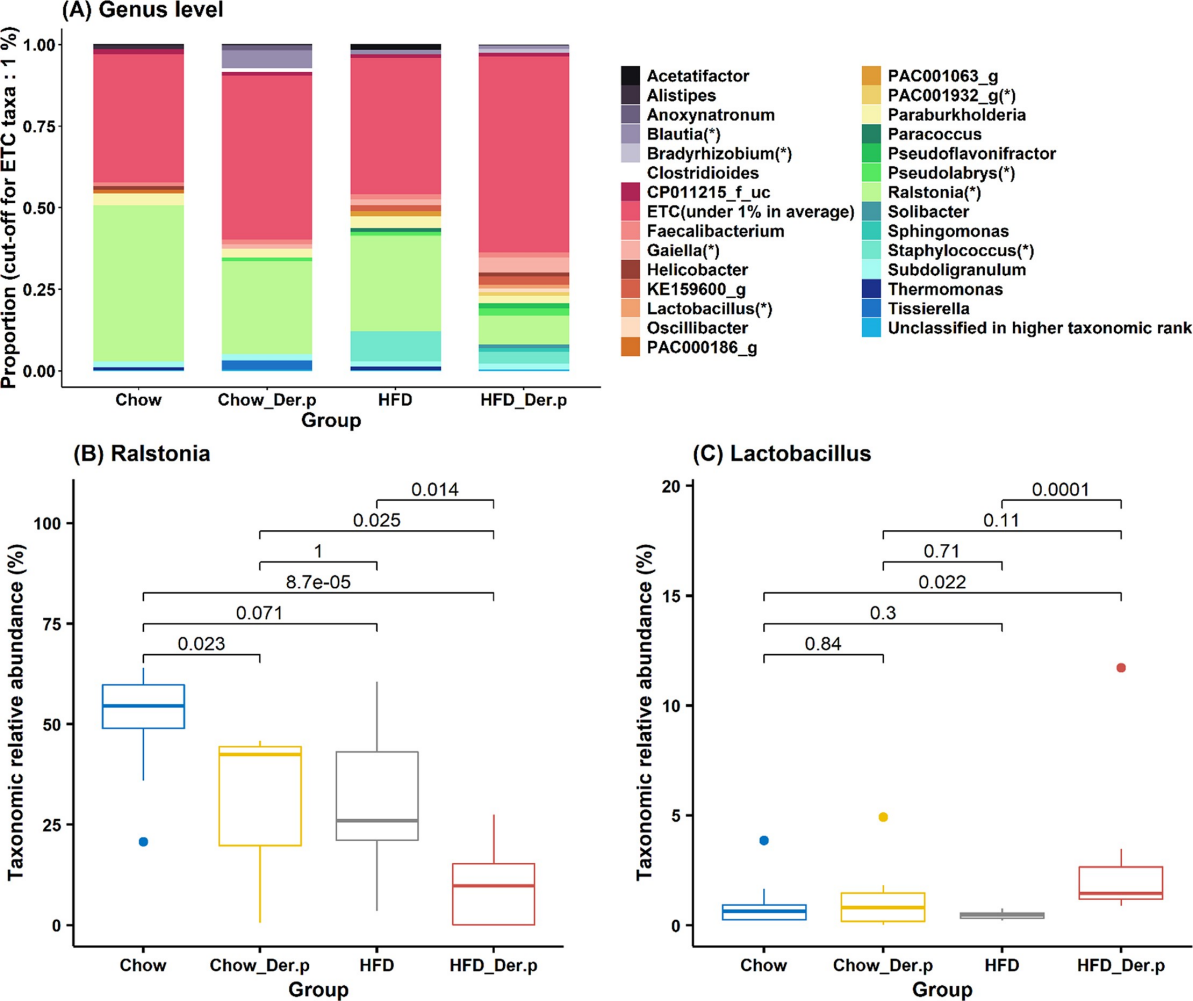
The relative abundances of bacteria at a genus level in Chow (n = 9), Chow_Der.p (n = 7), HFD (n = 7), and HFD_Der.p (n = 10) groups. (A) Bar chart of taxonomic proportions at a genus level. ETC taxa (under 1% in average) were not considered for comparison between groups. The asterisk (*) indicates statistically significant bacteria (Wilcoxon rank-sum test, p<0.05) for at least one group compared with other groups. Boxplot for the relative abundances of (B) *Ralstonia* and (C) *Lactobacillus* at a genus level with p-values (Wilcoxon rank-sum test). The median (line within the box), lower quartiles (Q1), upper quartiles (Q3), non-outlier range (whiskers), and outliers (dot) was described in the boxplot.

To identify how the Der.p exposure in both Chow and HFD samples affects the lung microbial environment, taxonomic relative abundances were compared at genus and species levels. The comparison of taxonomic relative abundances was restricted to each taxa covering more than 1%. At a genus level, the taxonomic relative abundance of *Ralstonia* showed a significant decrease in the HFD_Der.p group, as compared to the Chow (p = 8.7e-5), the Chow_Der.p (p = 0.025), and the HFD (p = 0.014) groups (Fig 3B).

**Fig 4:**
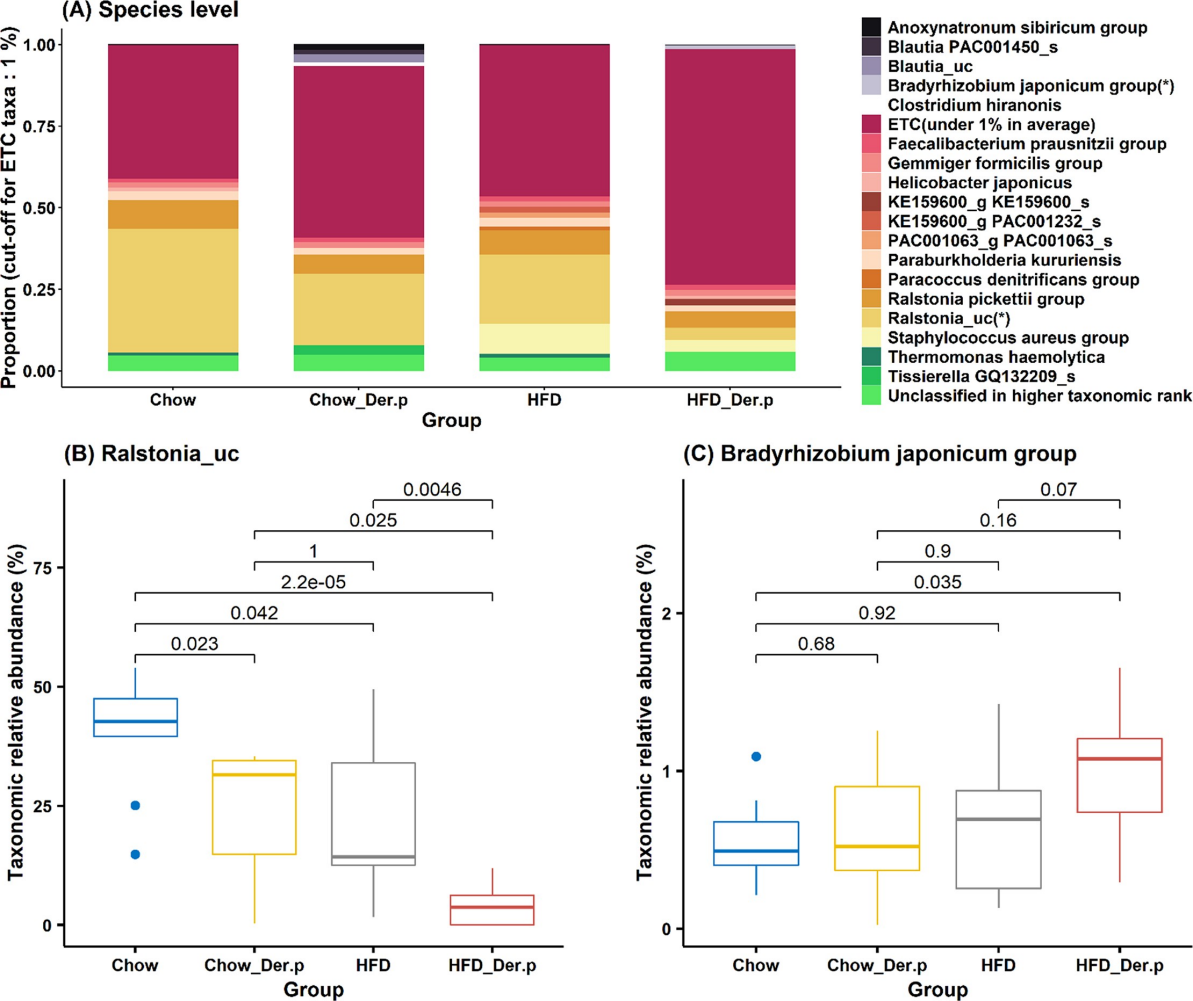
The relative abundances of bacteria at a species level in Chow (n = 9), Chow_Der.p (n = 7), HFD (n = 7), and HFD_Der.p (n = 10) groups. (A) Bar chart of taxonomic proportions at a species level. ETC taxa (under 1% in average) were not considered for comparison between groups. The asterisk (*) indicates statistically significant bacteria (Wilcoxon rank-sum test, p<0.05) for at least one group compared with other groups. Boxplot for the relative abundances of (B) *Ralstonia_u*c and (C) *Bradyrhizobium japonicum group* at the species level with p-values (Wilcoxon rank-sum test). The median (line within the box), lower quartiles (Q1), upper quartiles (Q3), non-outlier range (whiskers), and outliers (dot) was described in the boxplot.

Similarly, *Ralstonia_uc* (unclassified) was significantly reduced in the HFD_Der.p group, as compared to the Chow (p = 2.2e-5), the Chow_Der.p (p = 0.025), and the HFD (p = 0.0046) groups at a species level (Fig 4B).

On the other hand, the taxonomic relative abundance of *Lactobacillus* was markedly augmented in the HFD_Der.p group, as compared to the Chow (p = 0.022) and the HFD (p = 0.001) (Fig 3C) groups. Additionally, an overall increased abundance of *Pseudolabrys* (Chow, p = 0.041), *Gaiella* (Chow, p = 0.022), *Staphylococcus* (Chow_Der.p, p = 0.019), *PAC001932_g* (Chow, p = 0.022; Chow_Der.p, p = 0.032; HFD, p = 0.04), and *Bradyrhizobium* (Chow, p = 0.034) was identified in the HFD_Der.p group, except for *Blautia* (S2 Fig).

Also, an elevated expression of *Bradyrhizobium japonicum group* was detected in the HFD_Der.p versus the Chow (p = 0.035) group at a species level (Fig 4C). These results suggest that the fluctuations of taxonomic composition may occur in the HFD mouse’s lung after Der.p exposure.

### Alterations of bacterial species richness and diversity in groups

**Fig 5:**
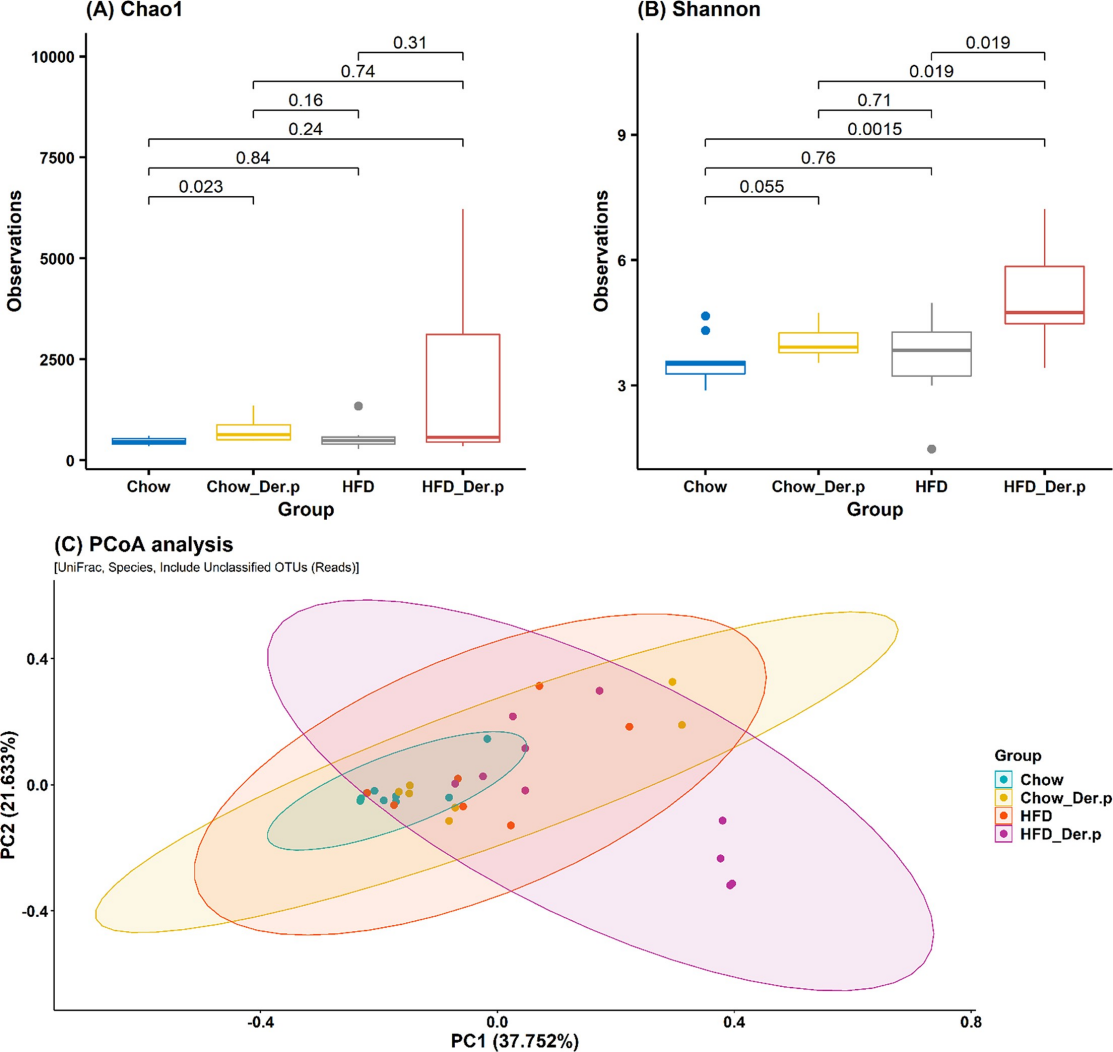
Alpha and beta diversity in lung microbiome in Chow (n = 9), Chow_Der.p (n = 7), HFD (n = 7), and HFD_Der.p groups (n = 10). The boxplot of (A) Chao1, (B) Shannon index was shown with p-values (Wilcoxon rank-sum test). The median (line within the box), lower quartiles (Q1), upper quartiles (Q3), non-outlier range (whiskers), and outliers (dot) was described in the boxplot. (C) The PCoA analysis was fulfilled at a species level with UniFrac distances, including unclassified OTUs.

The richness of bacterial species was investigated through Chao1, ACE, Jackknife, and the number of OTUs at a species level between groups. Observation values of Chao1 were enhanced in the Chow_Der.p compared with the Chow (p = 0.023) (Fig 5A).

Other indexes related to species richness were also increased in the Chow_Der.p versus the Chow (S3 Fig).

The bacterial diversity was estimated via Shannon, Simpson, and phylogenetic indexes at a species level (S3 Fig). Observation values of the Shannon index were markedly increased in the HFD_Der.p group, as compared to the Chow (p = 0.0015), the Chow_Der.p (p = 0.019), and the HFD (p = 0.019) (Fig 5B) groups. In contrast, Simpson’s observation values were reduced in the HFD_Der.p group, as compared to the Chow (p = 0.006), the Chow_Der.p (p = 0.008), and the HFD (p = 0.006) groups (S3 Fig). Considering species evenness and richness, these results imply that Der.p exposure results in the changes in the bacterial diversity of the HFD mouse’s lung.

### Similarities of bacterial community composition between samples

The PCoA was performed to uncover distribution of bacterial communities in samples. At a species level, UniFrac distances were measured for the PCoA, including unclassified OTUs. The PCoA result showed that the HFD_Der.p group was widely distributed with distinct patterns, as compared to other groups (Fig 5C). This result indicates that samples in the HFD_Der.p group shared similar bacterial composition, compared to other groups. Also, the UPGMA clustering was fulfilled to identify hierarchical groups in samples. We found that samples were clustered according to their group (S4 Fig).

### Functional prediction based on the 16S rRNA data

**Fig 6:**
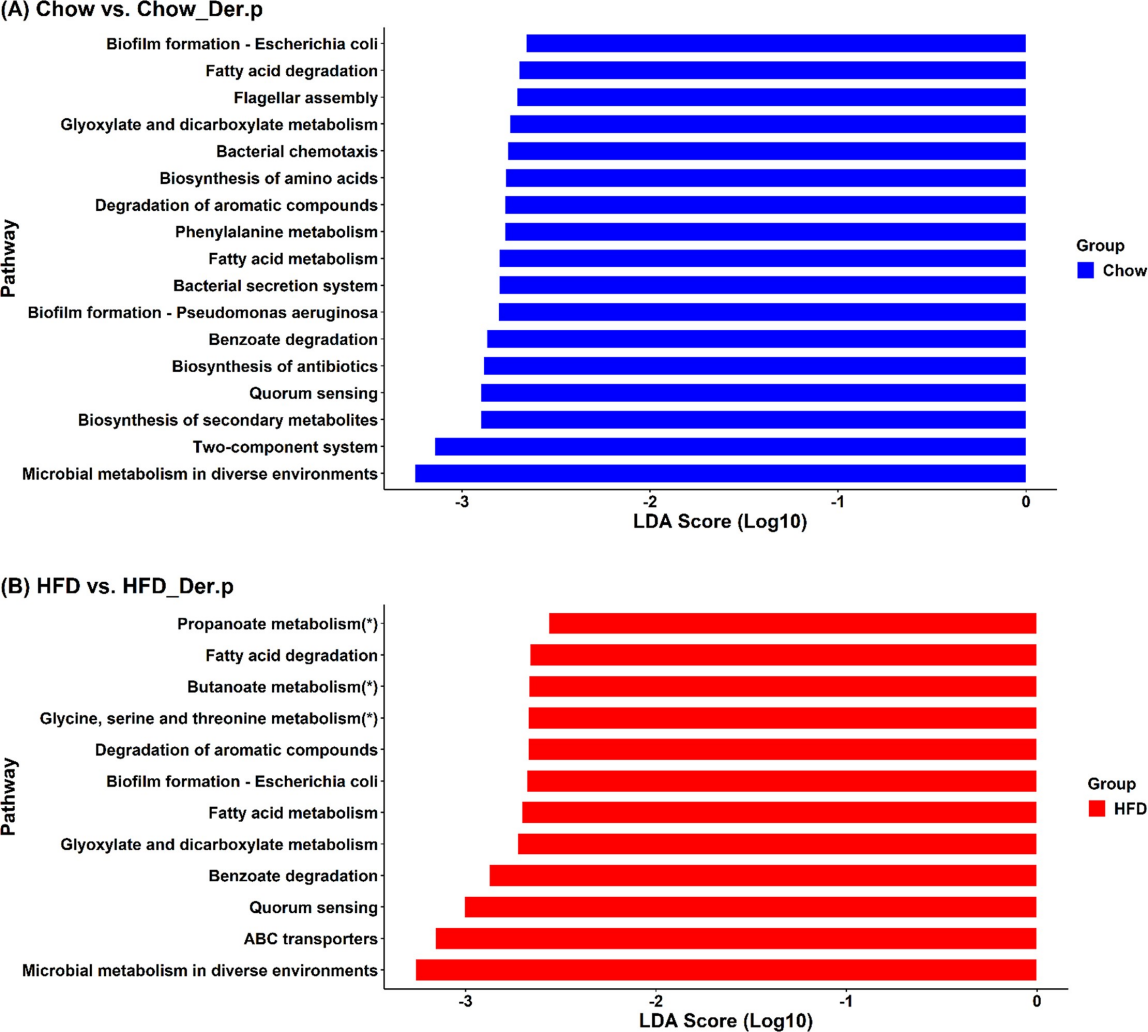
Predicted metabolic signatures based on 16S rRNA data. PICRUSt and LEfSe analyses were performed to predict functional potential within KEGG categories. The cut-off of LDA scores is 2 and p<0.05. The asterisk (*) indicates pathways identified only in the HFD group against the Chow group. (A) Chow (n = 9) group versus Chow_Der.p (n = 7) group, (B) HFD (n = 7) group versus HFD_Der.p (n = 10) group.

To estimate how taxonomic differences of microbiome in both Chow and HFD groups after Der.p exposure affect their metabolic potential, KEGG-based functional profiles derived from 16S rRNA data were predicted using PICRUSt. We performed a PICRUSt analysis in the Chow group versus the Chow group exposed to allergens as well as in the HFD mouse group versus the HFD group exposed to allergens. A LEfSe analysis was then executed on PICRUSt results at KEGG pathway levels, and we observed their functional similarities and differences. LEfSe results showed a concurrent enrichment in fatty acid degradation, degradation of aromatic compounds, biofilm formation-Escherichia coli, fatty acid metabolism, glyoxylate and dicarboxylate metabolism, benzoate degradation, quorum sensing, and microbial metabolism in diverse environments in both Chow and HFD groups (Fig 6).

These results indicate that the Der.p exposure exhibits similar asthma symptoms regardless of obesity. Notably, the metabolic signatures related to macrophages such as propanoate metabolism, butanoate metabolism, and glycine-serine-threonine metabolism were exclusively identified in the HFD group (Fig 6B) versus the Chow group (Fig 6A). These results indicate that HFD-induced obesity could lead to abnormal macrophage activations in the lung after allergen exposure, which could exacerbate asthma symptoms related to immunity and immune responses.

## Discussion

The distribution of lung microbiota exerts a strong influence on shaping asthma phenotypes [32]. Accumulating evidence suggests that the imbalance of bacterial communities in the lung leads to different degrees of severity of asthma [33–35]. In this study, we found that dust mites (Der.p), the most common asthma trigger of inducing asthma pathogenesis, change lung microbial circumstances that regulate specific bacterial abundances. First, in the estimation of HFD-induced obesity, the body weight of HFD groups were significantly increased, as compared to Chow groups (Fig 2A and 2D).

In the evaluation of the allergen mouse model through allergen-induced immune markers such as Der.p-specific IgE and total IgE, the increases of both Der.p-specific IgE (Fig 2F) and total IgE levels (Fig 2E) were observed in both Chow and HFD groups after Der.p exposure. Based on these observations, this study confirms that the HFD mouse model and the well-established allergen mouse model are induced by Der.p sensitization and challenge.

Next, we discovered that the bacteria in genus and species levels are relatively differentiated among Chow, Chow_Der.p, HFD, and HFD_Der.p groups. In the HFD_Der.p group, *Ralstonia* and *Ralstonia_uc* were less abundant than other groups at genus and species levels (Figs 3B and 4B). The previous study explained that *Ralstonia*, formerly called *Pseudomonas* and *Pseudomonas aeruginosa*, is considered to be potentially pathogenic bacteria on neutrophilic asthma [36]. The researchers cultured bacteria from the sputum of subjects with stable asthma and then discovered a remarkable association among *Pseudomonas aeruginosa*, total cell counts, and the proportion and number of neutrophils. This result indicates that *Pseudomonas* plays a pathogenic role in potentiating asthma. On the other hand, *Lactobacillus* in the HFD_Der.p group exhibited more abundance than the Chow and HFD groups at a genus level (Fig 3C). A few studies demonstrated that *Lactobacillus* has protective effects to prevent asthma [37, 38]. In addition, Huang and colleagues discovered that children administered with *Lactobacillus paracasei*, *Lactobacillus fermentum*, and their combination show an attenuated severity of asthma [39]. Based on these results, we suggest that an increased abundance of *Lactobacillus* in the HFD_Der.p group may lead to a declining exacerbation of obesity-related asthma. *Staphylococcus* was exclusively increased in the HFD_Der.p group versus the Chow_Der.p group (S2 Fig). More recently, in a meta-analysis that has combined the results from 16 culture method studies, nasal *Staphylococcus* colonization revealed positive associations against asthma prevalence [40]. Although longitudinal cohort studies are necessary to confirm causalities between *Staphylococcus* and asthma, *Staphylococcus* needs to be considered a promising risk factor for asthma.

Through PICRUSt and LEfSe analyses, we compared the metabolic signatures of the Chow group with those of the Chow_Der.p group. We also compared the metabolic signatures of the HFD group with those of the HFD_Der.p group. This way, we predicted functional metabolisms in both Chow and HFD groups. In both Chow and HFD groups, various asthma-related fatty acid metabolisms occurring under allergen exposure were concurrently identified. Such metabolisms include fatty acid degradation, fatty acid metabolism, and glyoxylate and dicarboxylate metabolism (Fig 6). Fatty acids played a vital role in the production of inflammatory mediators concerning asthma pathogenesis [41], and asthma-related symptoms were identified in several observational studies [42–44]. Benzoate degradation, biofilm formation, and quorum sensing also explained relevance to asthma [45, 46]. These results suggest that concurrent metabolic signatures between Chow and HFD groups exposed to allergens could elucidate asthma pathogenesis regardless of obesity.

Importantly, butanoate, propanoate, and glycine-serine-threonin metabolisms were only identified in the HFD group, whereas such metabolisms were not found in the Chow group exposed to Der.p (Fig 6B). These metabolisms are involved in macrophages as pivotal regulators of immunity and inflammation in asthma, and influence the severity of allergic inflammation. First, propanoate levels, also called propionate, regulate lung immune responses, and the augmentation of propionate reduces lung inflammation [47]. Trompette and colleagues showed that short-chain fatty acids (SCFAs) propionate could induce immunological responses in the lung and affect airway diseases [48]. Second, an increase of butanoate, also called butyrate, might reinforce host defense without tissue-damaging inflammation, and butyrate-induced antimicrobial activity resulted in changes of macrophage metabolism [49]. Third, the necessity of serine metabolism was verified for macrophage interleukin (IL)-1 beta production [50]. Macrophage dysfunction showed heterogeneity and higher prevalence in asthma, which might affect diverse inflammatory phenotypes of asthma [51]. These results show that metabolic signatures related to HFD-induced obesity could affect the exacerbation of asthma in a state of allergen exposure. Additionally, flow cytometric analysis was performed to supplement these results. The proportion of macrophage phenotype showed a significant change in which M1 macrophages increased and M2 and aveloar macrophages decreased in the HFD group (S1 Fig). Thus, we need to consider obesity as a risk factor for causing malfunctions of lung macrophages. Most recently, accumulations of adipose tissues were identified within the airway wall of obese asthmatics. This shows a positive correlation between airway wall thickness and airway inflammation [52]. From their observations, we suggest that lung fat accrual of an allergen mouse model causes the abnormalities of lung macrophages, and thus enhances the chance of developing severe asthma.

There are a few limitations to our study. First, we estimated taxonomic relative abundances between groups, and predicted their metabolic potential based on 16S rRNA gene data. Although 16S rRNA gene sequencing unravels phylogenetic characteristics through a bacterial census, shotgun metagenome sequencing is required to survey comprehensive lung microbes such as viruses, fungi, yeasts, protists, and enzymes as well as to explore their actual functionalities rather than predicted. Second, we conducted this study using only C57BL/6 male mouse to uncover pathophysiological mechanisms in obese allergic asthma; however, our mouse model is restricted in mimicking all aspects and phenotypes of asthmatics [53], and several studies suggest that both sex and genetic background should be considered in microbial community studies [54, 55]. Therefore, additional experiments using diverse asthma mouse models are required to investigate whether obesity affects microbiota composition on diverse asthma subtypes. Third, treatment of antibiotics may reduce the cultivable bacteria in the respiratory tract, but it may also reduce the myeloid cell population in systemic sites similar to that found in germ-free mice, so more attention should be paid to the interpretation of the results [56]. In addition, samples from the lung can contain relatively low biomass [57], in contrast to fecal samples, and inclusion of extensive controls against contamination at many levels of the process is advisable [58]. We have tried to minimize the risk of contamination and false-positive results by using negative controls in several processes, but there are limitations.

Based on comprehensive microbiota analysis reflecting the phenotypes of obese asthma, we could establish functional roles of microbiome in obese asthma. Our study shows that abundance levels of a specific lung microbiome were differentiated between Chow and HFD groups exposed to Der.p, and unveiled metabolic signatures related to obese allergic asthma. These results offer an opportunity to comprehend a microbiome community of obese allergic asthma, and shed light on the functional roles of lung microbiota inducing the pathogenesis of severe asthma.

## Supporting information

S1 Fig Lung histopathology and proportions of macrophage phenotypes of lung tissues. (TIF)

S2 Fig Additional six bacteria showing differences of taxonomic relative abundances in Chow, Chow_Der.p, HFD, and HFD_Der.p groups at a genus level. (PDF)

S3 Fig Chao1, ACE, Jackknife, and the number of OTUs at a species level between Chow, Chow_Der.p, HFD, and HFD_Der.p groups. (PDF)

S4 Fig UPGMA clustering with bacterial profiles of samples. (PDF)

S1 Table Composition of the normal diet (Chow) and high fat diet (HFD) fed to mice. (DOCX)
