# Supplementary figures and images for: Alterations of lung microbial communities in obese allergic asthma and metabolic potential
Source: PLoS One. 2021 Oct 28;16(10):e0256848. doi: 10.1371/journal.pone.0256848 (PMC8553092; doi:10.1371/journal.pone.0256848)

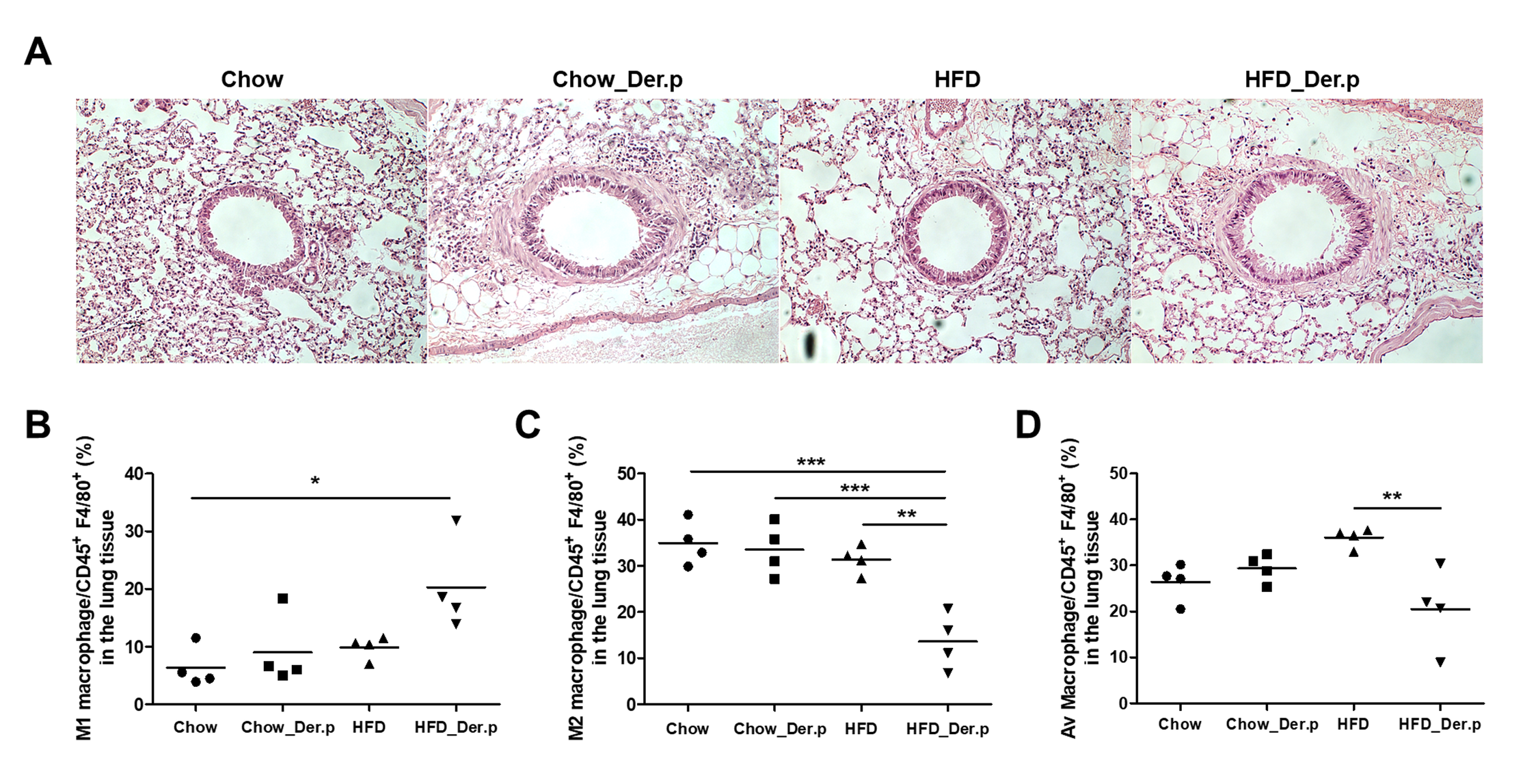

Supplement: S1 Fig — (TIF) [file pone.0256848.s001.tif]

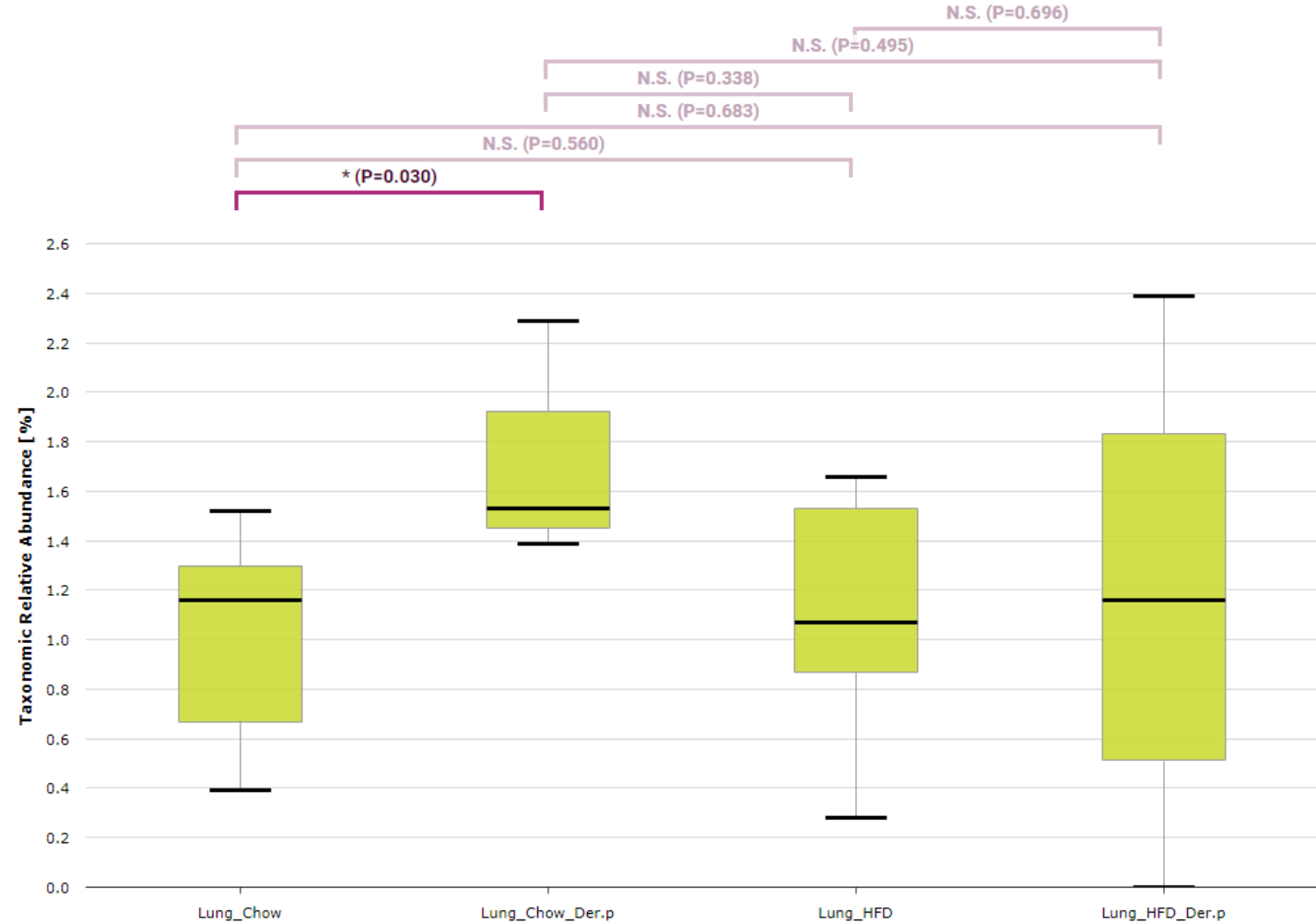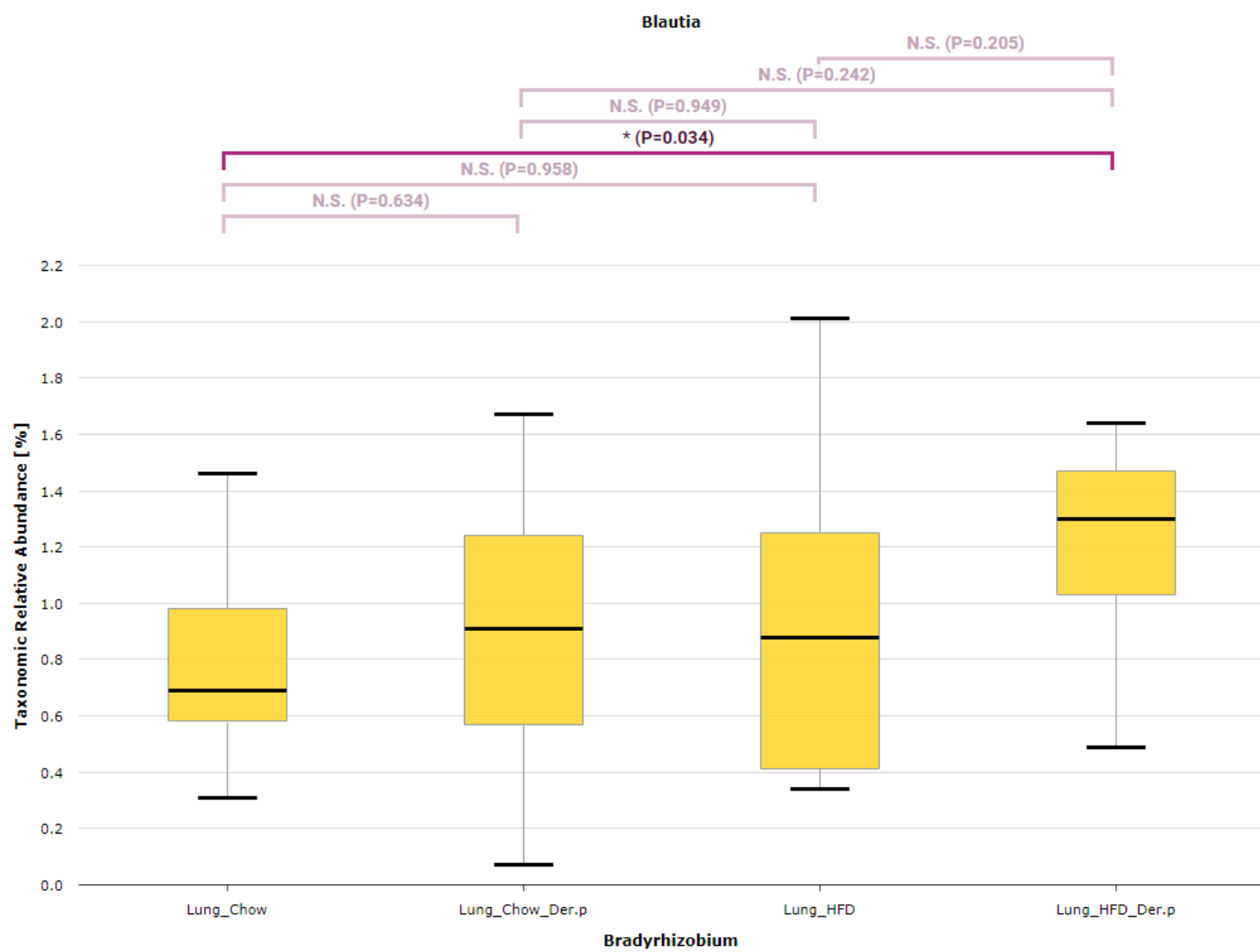

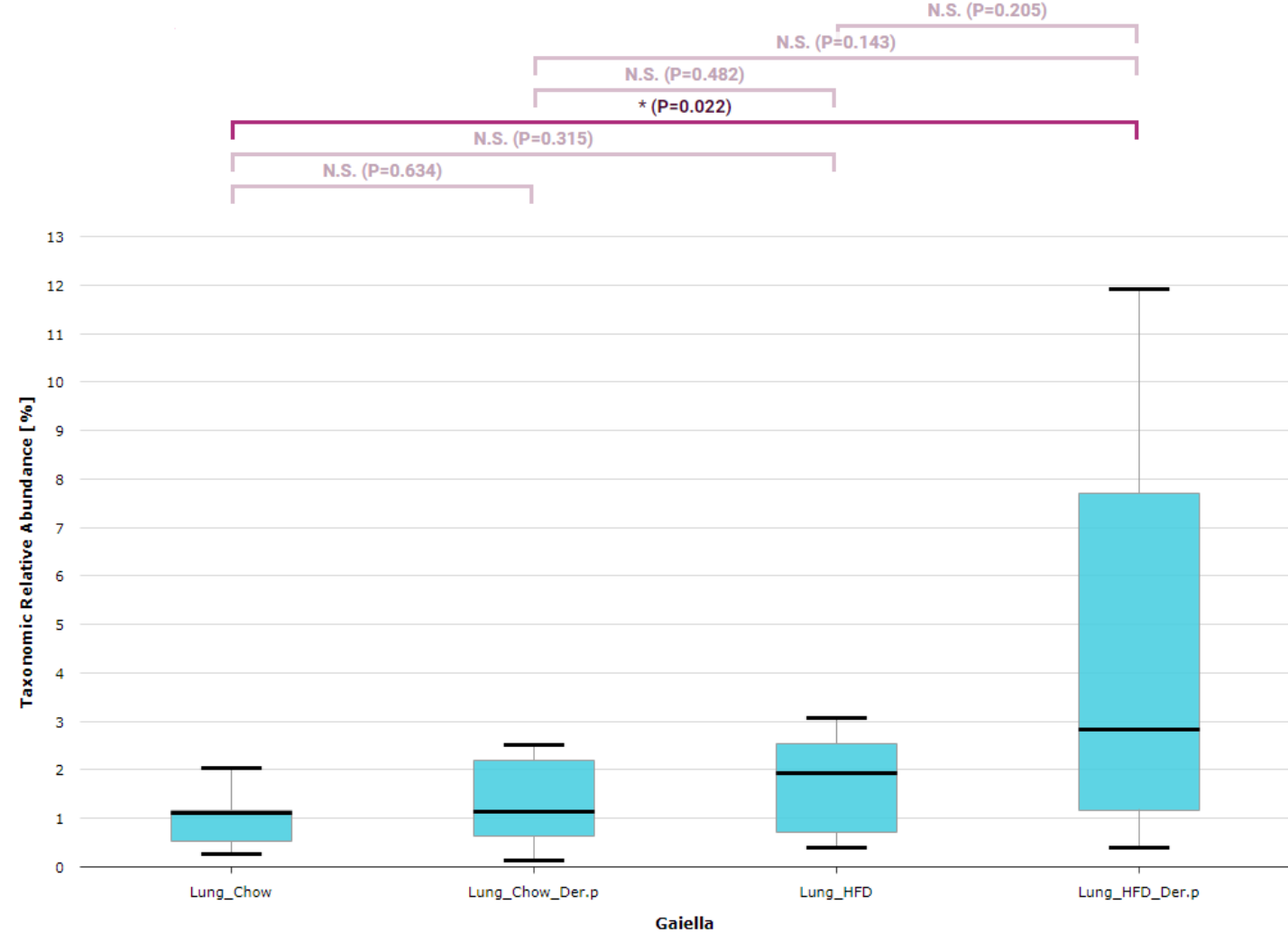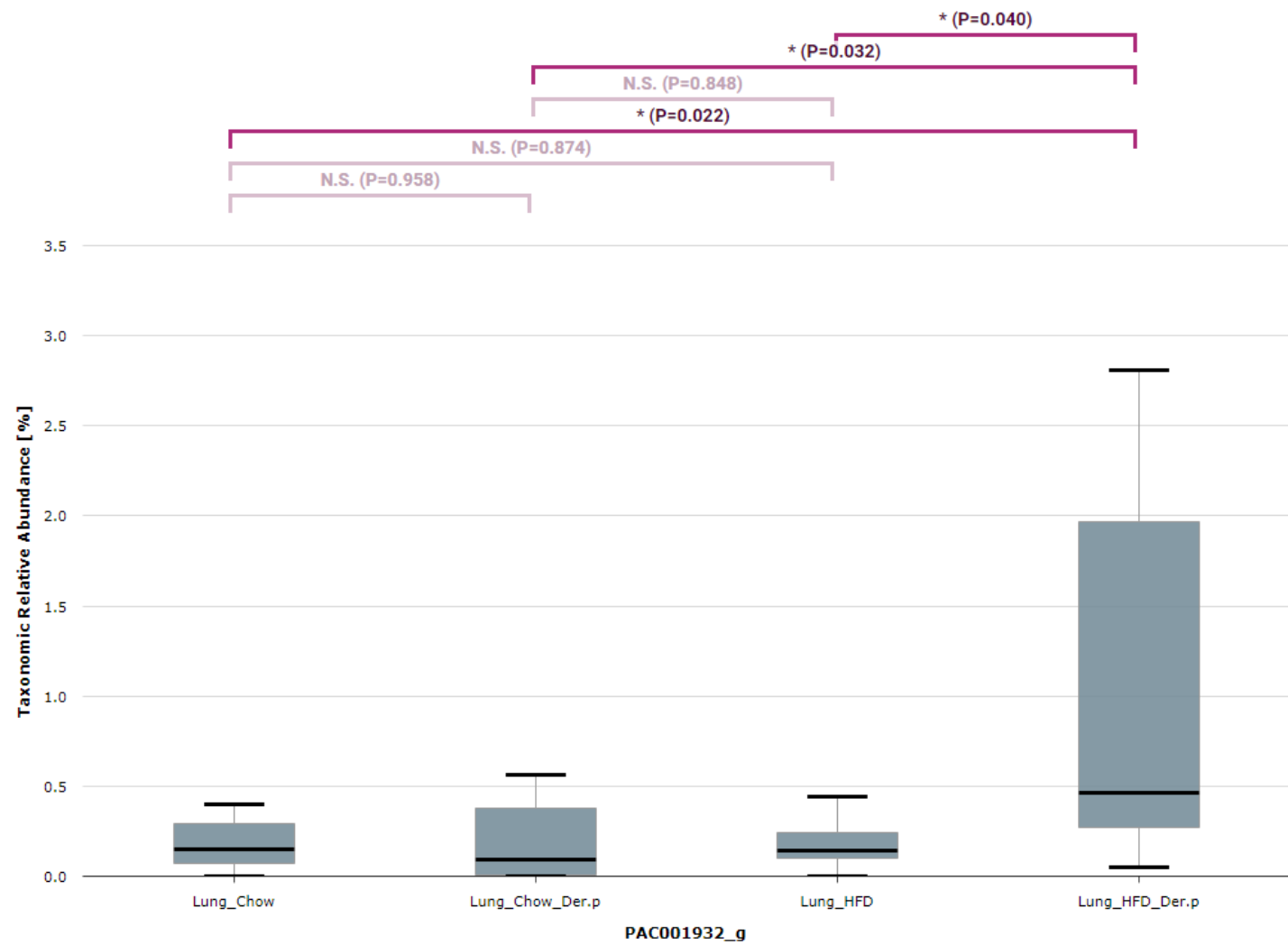

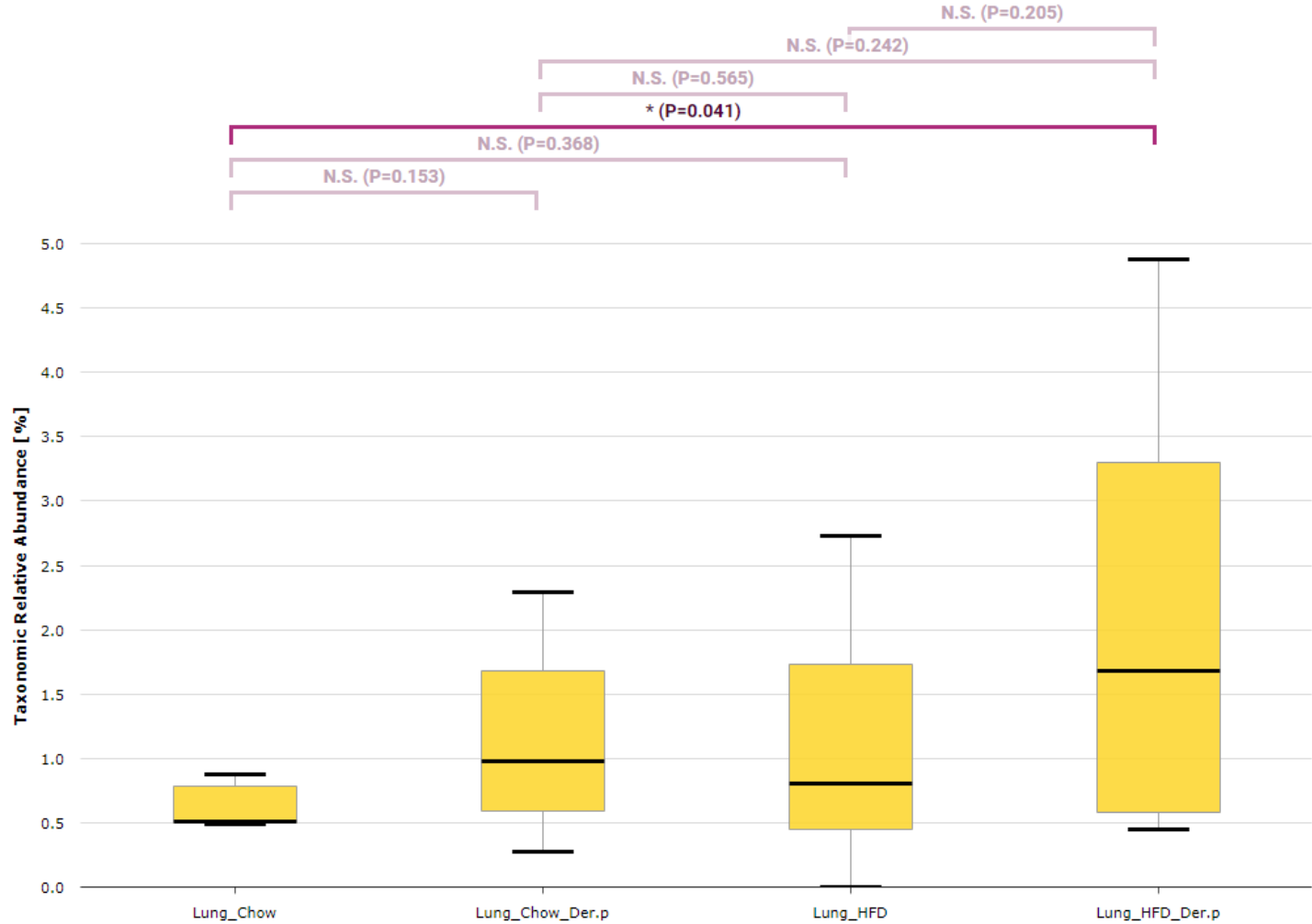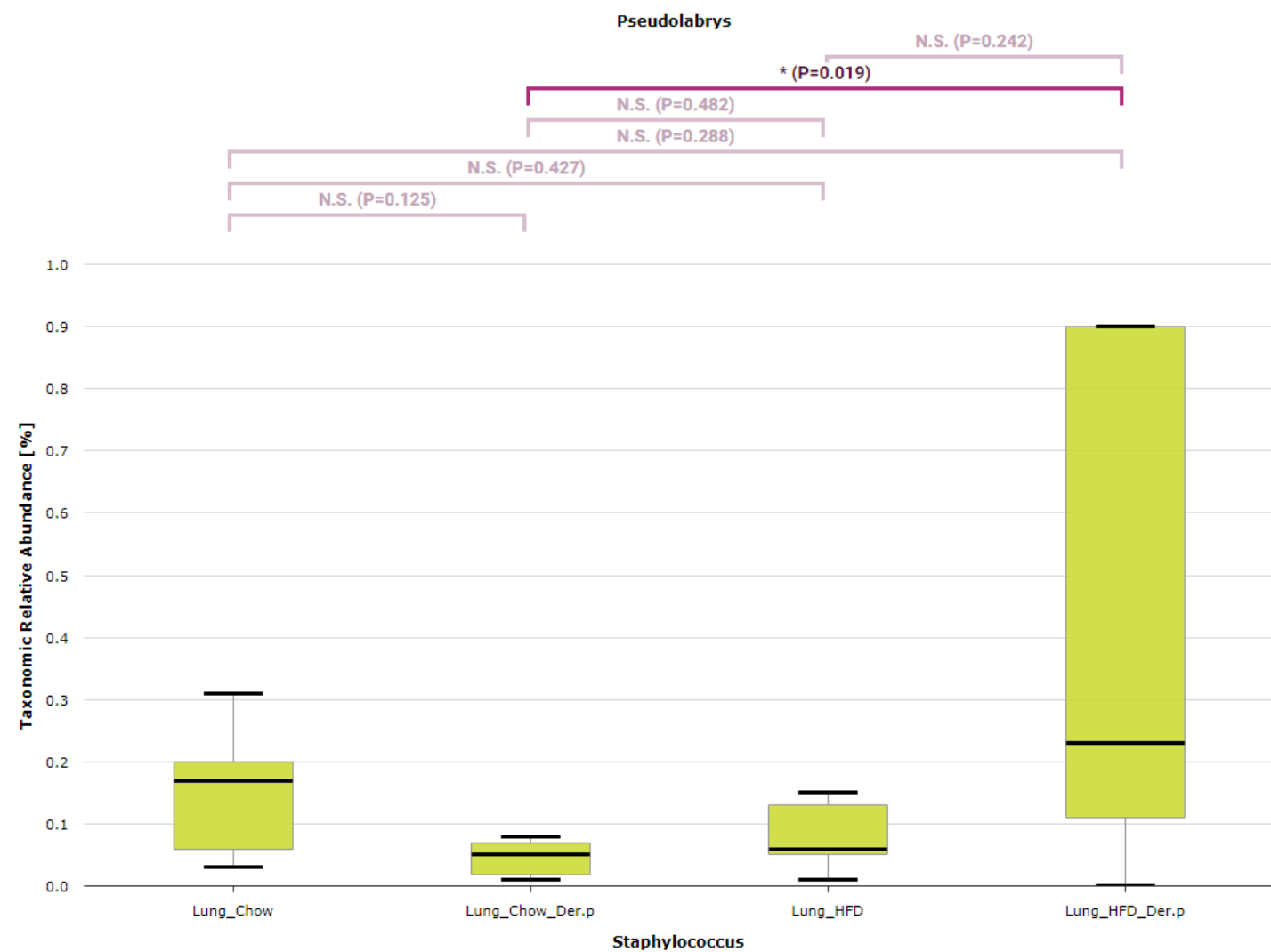

Supplement: S2 Fig — (PDF) [file pone.0256848.s002.pdf]

UPGMA Clustering

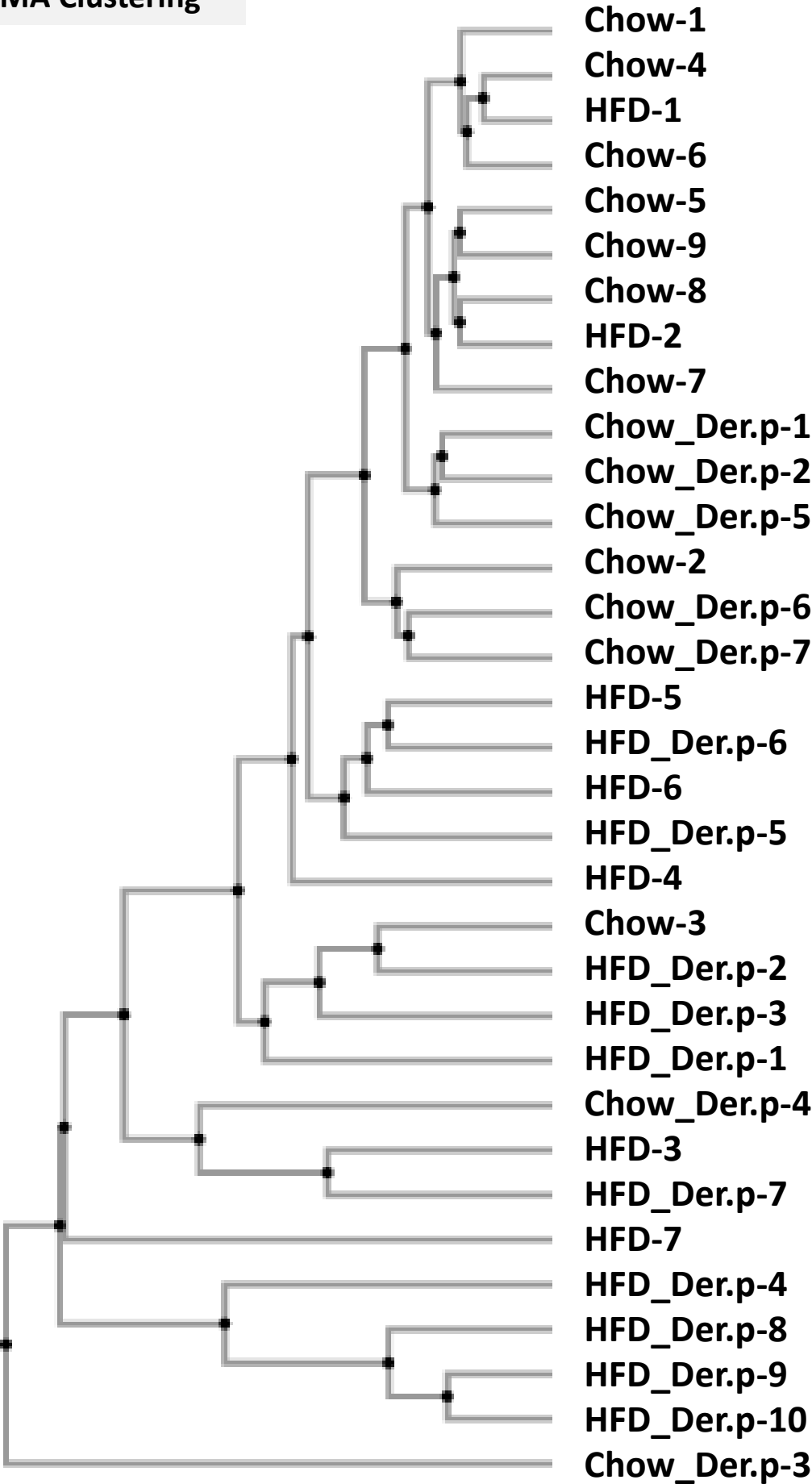

0.173

Supplement: S4 Fig — (PDF) [file pone.0256848.s004.pdf]
