# Supplementary material for: Alterations of lung microbial communities in obese allergic asthma and metabolic potential
Source: PLoS One. 2021 Oct 28;16(10):e0256848. doi: 10.1371/journal.pone.0256848 (PMC8553092; doi:10.1371/journal.pone.0256848)
Supplement: S3 Fig — (PDF) [file pone.0256848.s003.pdf]

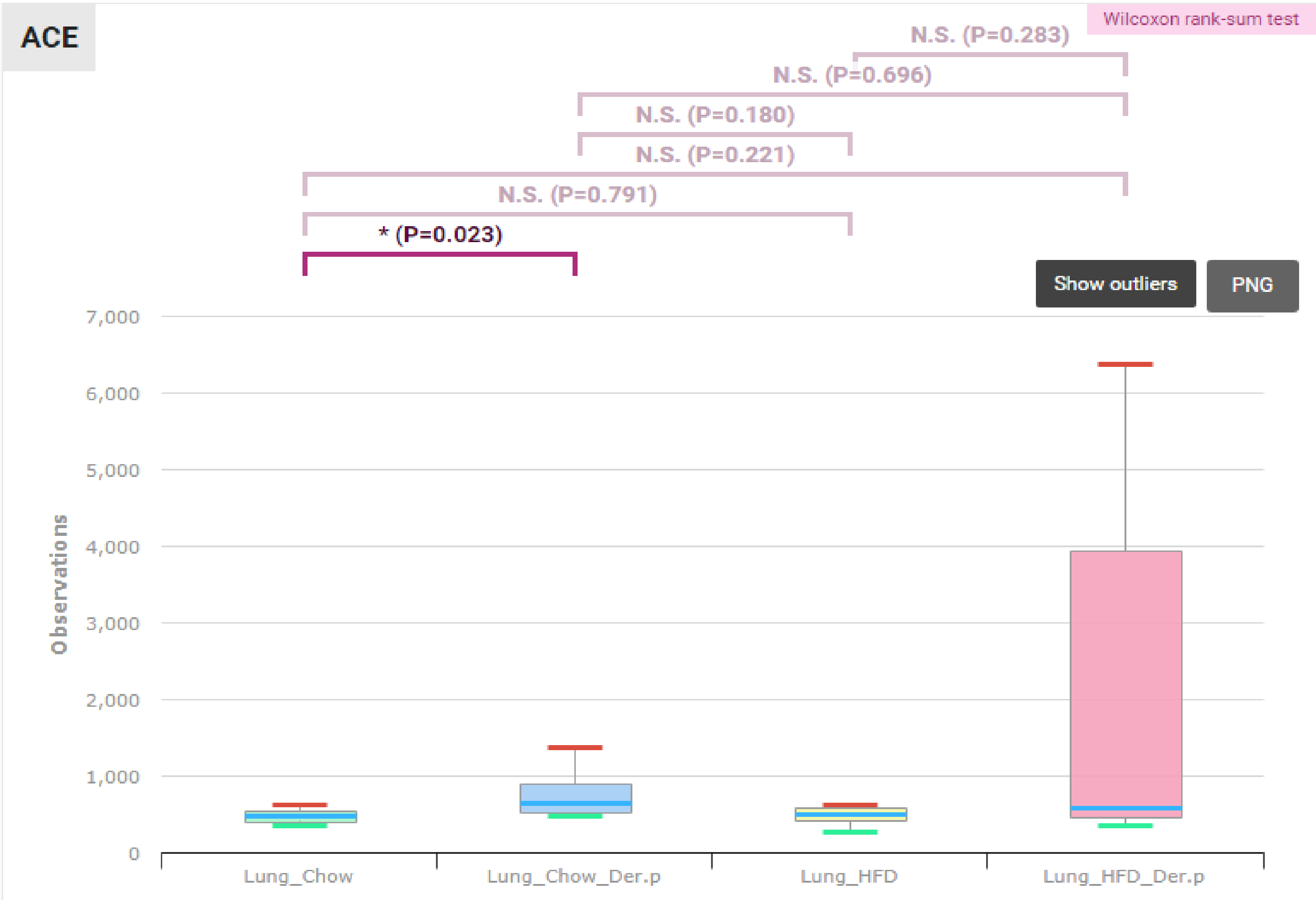
ACE
Wilcoxon rank-sum test
N.S. (P=0.283)
N.S. (P=0.696)
N.S. (P=0.180)
N.S. (P=0.221)
N.S. (P=0.791)
* (P=0.023)
Show outliers
PNG
Observations
7,000
6,000
5,000
4,000
3,000
2,000
1,000
0
Lung_Chow
Lung_Chow_Der.p
Lung_HFD
Lung_HFD_Der.p

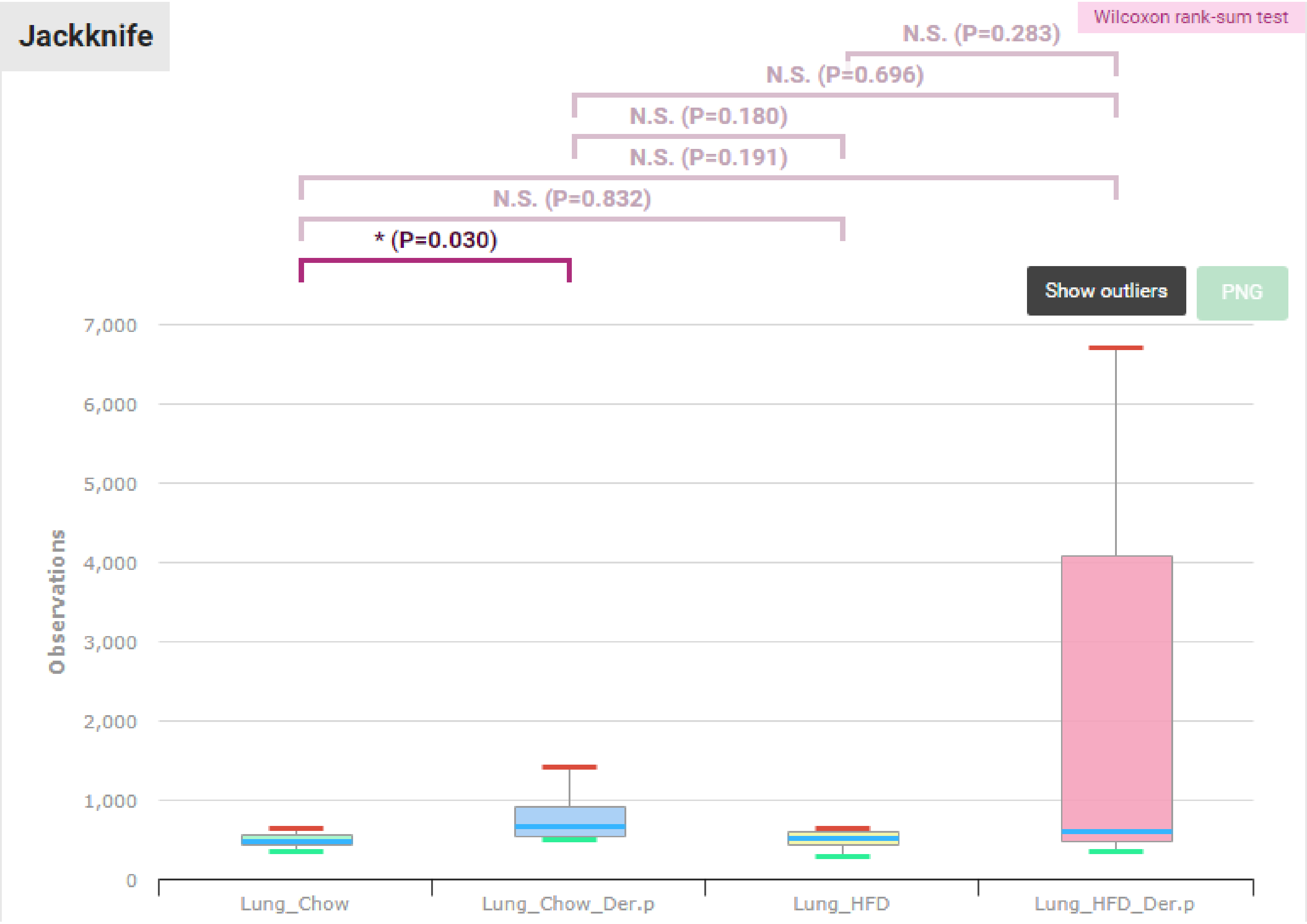
Jackknife
Wilcoxon rank-sum test
N.S. (P=0.283)
N.S. (P=0.696)
N.S. (P=0.180)
N.S. (P=0.191)
N.S. (P=0.832)
* (P=0.030)
Show outliers
PNG
Observations
7,000
6,000
5,000
4,000
3,000
2,000
1,000
0
Lung_Chow
Lung_Chow_Der.p
Lung_HFD
Lung_HFD_Der.p

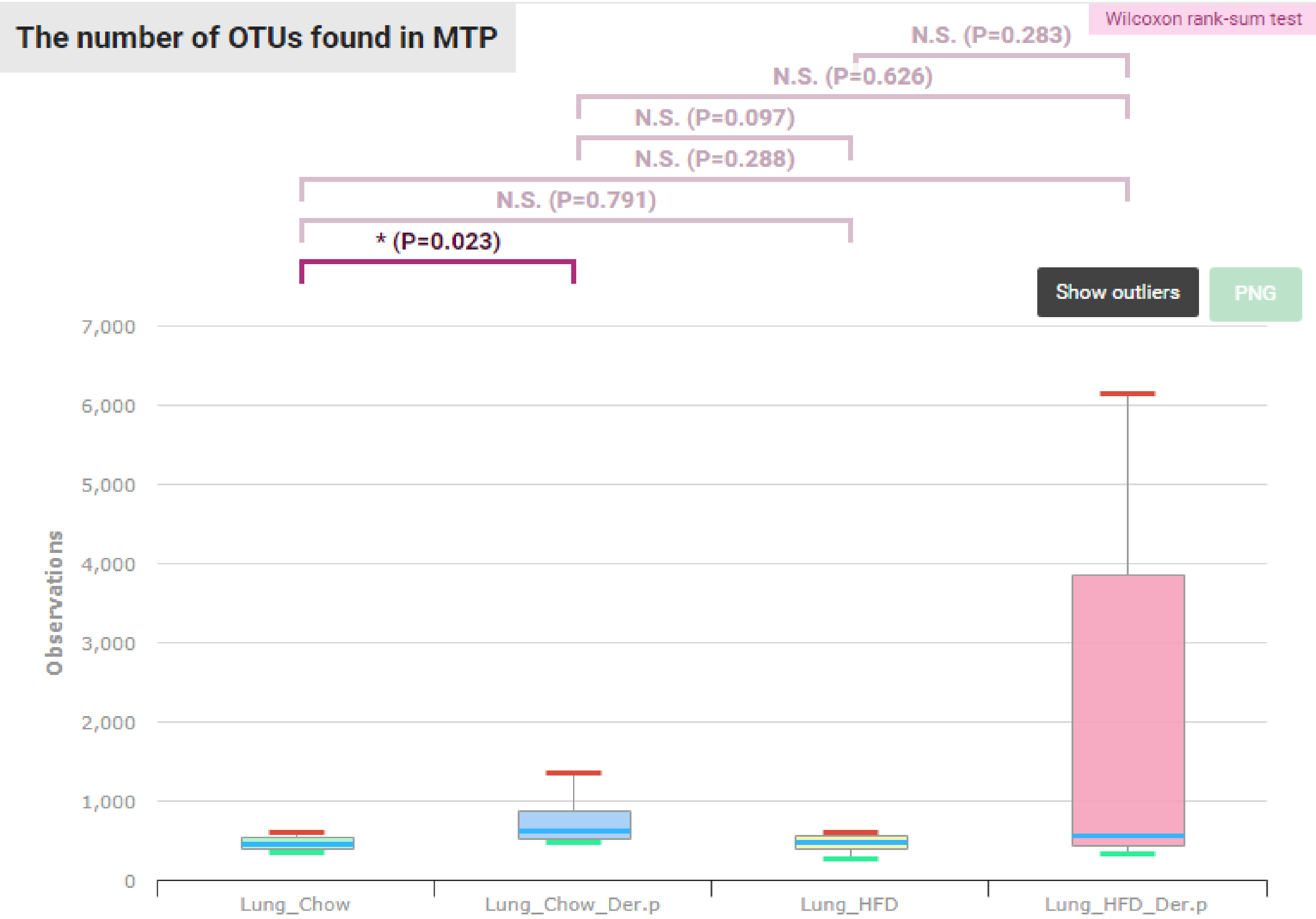
The number of OTUs found in MTP
Wilcoxon rank-sum test
N.S. (P=0.283)
N.S. (P=0.626)
N.S. (P=0.097)
N.S. (P=0.288)
N.S. (P=0.791)
* (P=0.023)
Show outliers
PNG
Observations
7,000
6,000
5,000
4,000
3,000
2,000
1,000
0
Lung_Chow
Lung_Chow_Der.p
Lung_HFD
Lung_HFD_Der.p

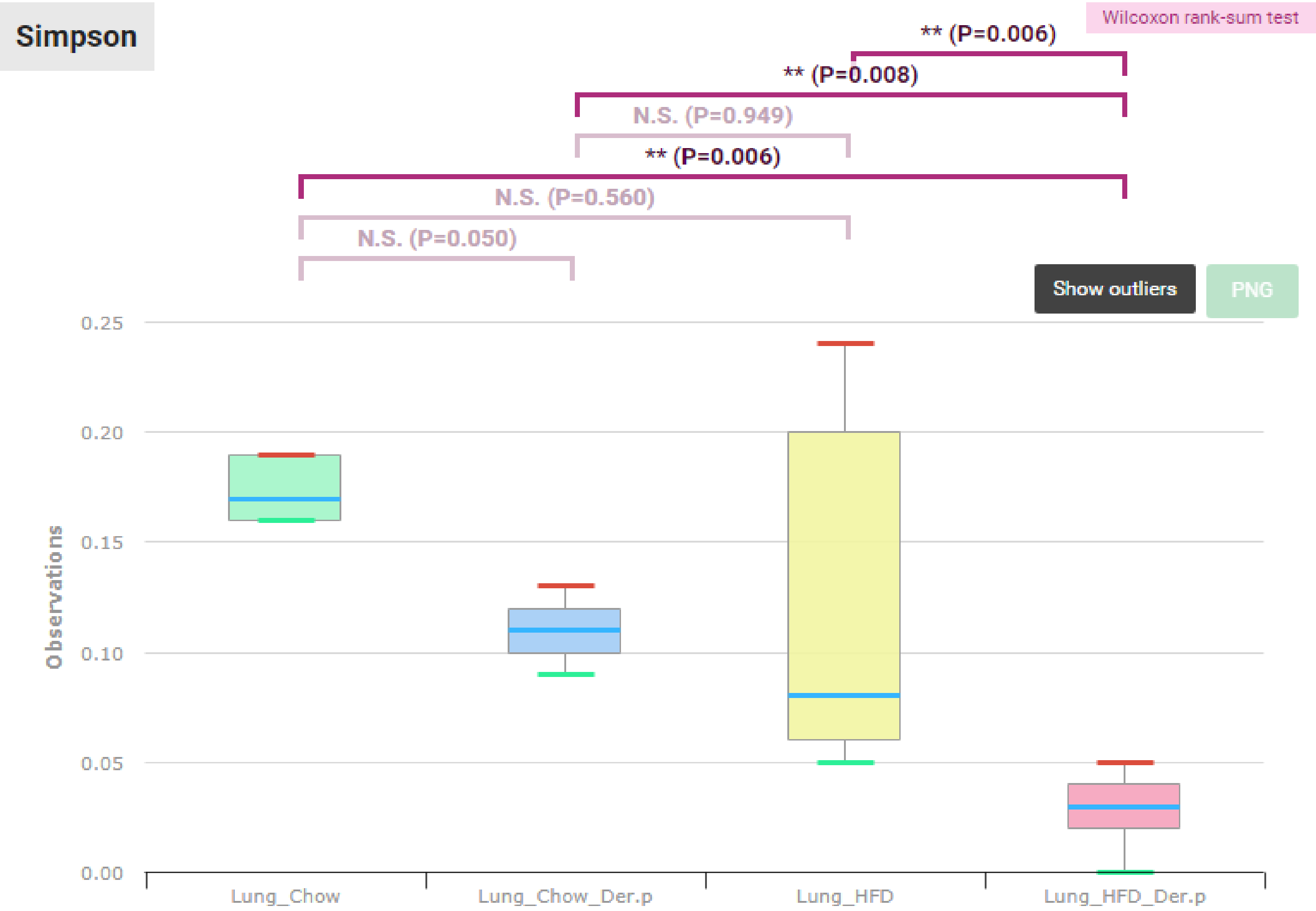

Simpson
Wilcoxon rank-sum test
** (P=0.006)
** (P=0.008)
N.S. (P=0.949)
** (P=0.006)
N.S. (P=0.560)
N.S. (P=0.050)
Show outliers
PNG
Observations
0.25
0.20
0.15
0.10
0.05
0.00
Lung_Chow
Lung_Chow_Der.p
Lung_HFD
Lung_HFD_Der.p

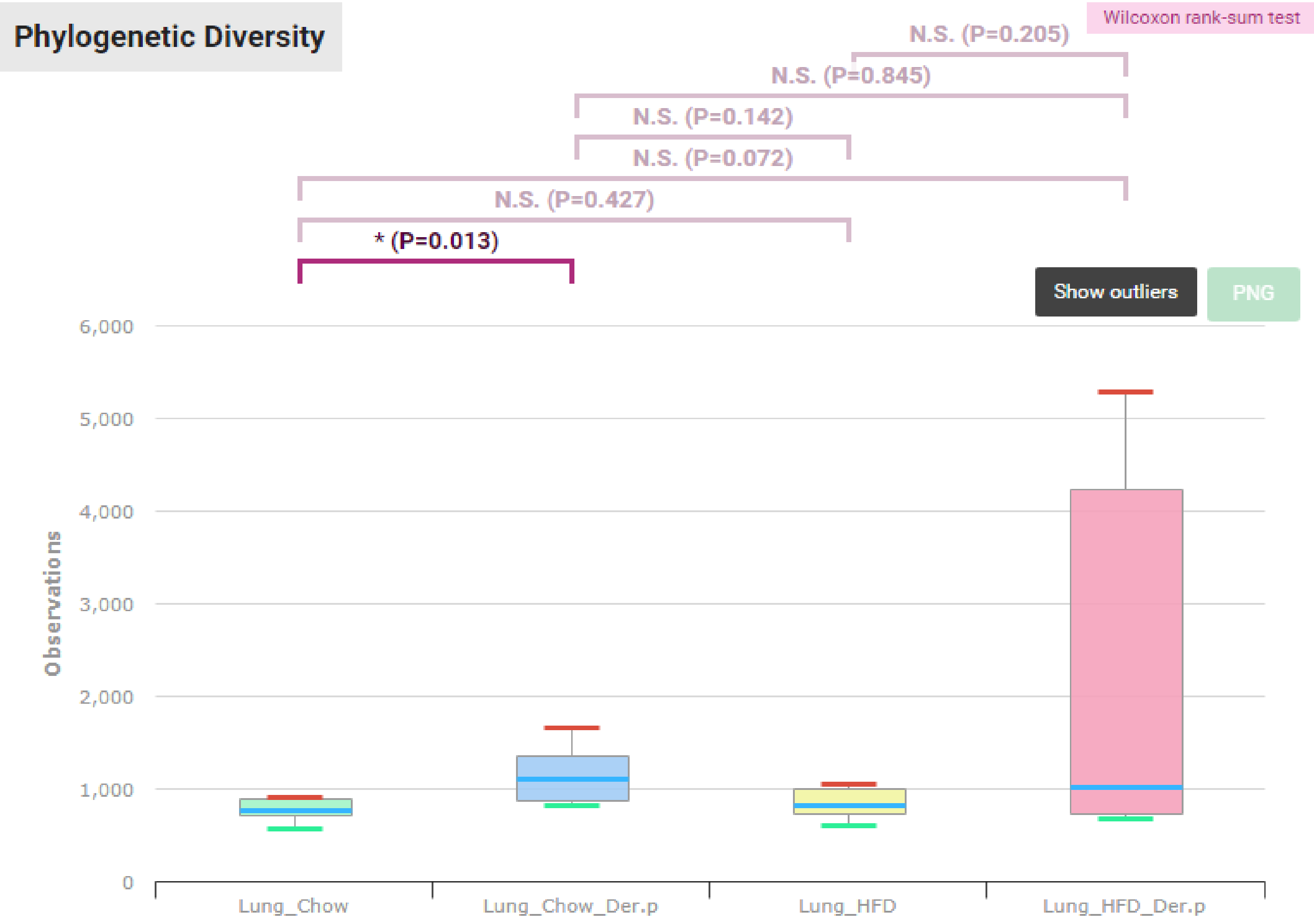

Phylogenetic Diversity
Wilcoxon rank-sum test
N.S. (P=0.205)
N.S. (P=0.845)
N.S. (P=0.142)
N.S. (P=0.072)
N.S. (P=0.427)
* (P=0.013)
Show outliers
PNG
Observations
6,000
5,000
4,000
3,000
2,000
1,000
0
Lung_Chow
Lung_Chow_Der.p
Lung_HFD
Lung_HFD_Der.p
