# Supplementary material for: Alterations of lung microbial communities in obese allergic asthma and metabolic potential
Source: PLoS One. 2021 Oct 28;16(10):e0256848. doi: 10.1371/journal.pone.0256848 (PMC8553092; doi:10.1371/journal.pone.0256848)
Supplement: S1 Table — (DOCX) [file pone.0256848.s005.docx]

**S1 Table. Composition of the normal diet (Chow) and high fat diet (HFD) fed to mice**

| Groups  (Research Diet No.) | **Chow**  **(D12450B)** | | **HFD**  **(D12451)** | |
| --- | --- | --- | --- | --- |
| Class description | gm% | kcal% | gm% | kcal% |
| Protein | 19.2 | 20 | 23.7 | 20 |
| Carbohydrate | 67.3 | 70 | 41.4 | 35 |
| Fat | 4.3 | 10 | 23.6 | 45 |
| Total |  | 100 |  | 100 |
| Kcal/gm | 3.85 |  | 4.73 |  |
| Ingredient | gm | kcal | gm | kcal |
| Casein, 30 Mesh | 200 | 800 | 200 | 800 |
| L-Cystine | 3 | 12 | 3 | 12 |
| Corn Starch | 315 | 1260 | 72.8 | 291 |
| Maltodextrin 10 | 35 | 140 | 100 | 400 |
| Sucrose | 350 | 1400 | 172.8 | 691 |
| Cellulose, BW200 | 50 | 0 | 50 | 0 |
| Soybene Oil | 25 | 225 | 25 | 225 |
| Lard | 20 | 180 | 177.5 | 1598 |
| Mineral Mix S10026 | 10 | 0 | 10 | 0 |
| DiCalcium Phosphate | 13 | 0 | 13 | 0 |
| Calcium Carbonate | 5.5 | 0 | 5.5 | 0 |
| Potassium Citrate, 1 H2O | 16.5 | 0 | 16.5 | 0 |
| Vitamin Mix V10001 | 10 | 40 | 10 | 40 |
| Choline Bitartrate | 2 | 0 | 2 | 0 |
| FD&C Yellow Dye#5 | 0.05 | 0 | 0 | 0 |
| FD&C Red Dye #40 | 0 | 0 | 0.05 | 0 |
| Total | 1055.05 | 4057 | 858.15 | 4057 |
